# Supplementary material for: Hybrid Models and Biological Model Reduction with PyDSTool
Source: PLoS Comput Biol. 2012 Aug 9;8(8):e1002628. doi: 10.1371/journal.pcbi.1002628 (PMC3415397; doi:10.1371/journal.pcbi.1002628)
Supplement: Text S4 — Complete source code for the PyDSTool package (version 0.88.120504). Includes API documentation and help files linking to web pages. This file is identical to the current public release on Sourceforge.net. (ZIP) [file pcbi.1002628.s004.zip › PyDSTool/html/PyDSTool.PyCont.ContClass'-module.html]

xml version="1.0" encoding="ascii"?


PyDSTool.PyCont.ContClass'


| Home | Trees | Indices | Help | PyDSTool |
| --- | --- | --- | --- | --- |

Package PyDSTool ::
Package PyCont ::
Module ContClass'

[hide private]

[frames] | no frames]

# Module ContClass'

source code

ContClass stores continuation curves for a specified model.

Drew LaMar, March 2006


|  |  |
| --- | --- |
| Classes | [hide private] |

ContClass  
Stores continuation curves for a specified model.


|  |  |
| --- | --- |
| Functions | [hide private] |

|  |  |
| --- | --- |
| beta(a, b, size=None)  The Beta distribution over ``[0, 1]``. | source code |

|  |  |
| --- | --- |
| binomial(n, p, size=None)  Draw samples from a binomial distribution. | source code |

|  |  |
| --- | --- |
| bytes(length)  Return random bytes. | source code |

|  |  |
| --- | --- |
| chisquare(df, size=None)  Draw samples from a chi-square distribution. | source code |

|  |  |
| --- | --- |
| distutil\_destination()  Makes the goofy destination directory string so that we can find where the distutils fortran compiler puts things. | source code |

|  |  |
| --- | --- |
| exponential(scale=1.0, size=None)  Exponential distribution. | source code |

|  |  |
| --- | --- |
| f(dfnum, dfden, size=None)  Draw samples from a F distribution. | source code |

|  |  |
| --- | --- |
| gamma(shape, scale=1.0, size=None)  Draw samples from a Gamma distribution. | source code |

|  |  |
| --- | --- |
| geometric(p, size=None)  Draw samples from the geometric distribution. | source code |

|  |  |
| --- | --- |
| get\_state()  Return a tuple representing the internal state of the generator. | source code |

|  |  |
| --- | --- |
| gumbel(loc=0.0, scale=1.0, size=None)  Gumbel distribution. | source code |

|  |  |
| --- | --- |
| hypergeometric(ngood, nbad, nsample, size=None)  Draw samples from a Hypergeometric distribution. | source code |

|  |  |
| --- | --- |
| laplace(loc=0.0, scale=1.0, size=None)  Draw samples from the Laplace or double exponential distribution with specified location (or mean) and scale (decay). | source code |

|  |  |
| --- | --- |
| logistic(loc=0.0, scale=1.0, size=None)  Draw samples from a Logistic distribution. | source code |

|  |  |
| --- | --- |
| lognormal(mean=0.0, sigma=1.0, size=None)  Return samples drawn from a log-normal distribution. | source code |

|  |  |
| --- | --- |
| logseries(p, size=None)  Draw samples from a Logarithmic Series distribution. | source code |

|  |  |
| --- | --- |
| multinomial(n, pvals, size=None)  Draw samples from a multinomial distribution. | source code |

|  |  |
| --- | --- |
| multivariate\_normal(mean, cov, size=...)  Draw random samples from a multivariate normal distribution. | source code |

|  |  |
| --- | --- |
| negative\_binomial(n, p, size=None)  Draw samples from a negative\_binomial distribution. | source code |

|  |  |
| --- | --- |
| noncentral\_chisquare(df, nonc, size=None)  Draw samples from a noncentral chi-square distribution. | source code |

|  |  |
| --- | --- |
| noncentral\_f(dfnum, dfden, nonc, size=None)  Draw samples from the noncentral F distribution. | source code |

|  |  |
| --- | --- |
| normal(loc=0.0, scale=1.0, size=None)  Draw random samples from a normal (Gaussian) distribution. | source code |

|  |  |
| --- | --- |
| pareto(a, size=None)  Draw samples from a Pareto distribution with specified shape. | source code |

|  |  |
| --- | --- |
| permutation(x)  Randomly permute a sequence, or return a permuted range. | source code |

|  |  |
| --- | --- |
| poisson(lam=1.0, size=None)  Draw samples from a Poisson distribution. | source code |

|  |  |
| --- | --- |
| power(a, size=None)  Draws samples in [0, 1] from a power distribution with positive exponent a - 1. | source code |

|  |  |
| --- | --- |
| rand(d0, d1, dn, ...)  Random values in a given shape. | source code |

|  |  |
| --- | --- |
| randint(low, high=None, size=None)  Return random integers from `low` (inclusive) to `high` (exclusive). | source code |

|  |  |
| --- | --- |
| randn(d1=..., dn=..., ...)  Return a sample (or samples) from the "standard normal" distribution. | source code |

|  |  |
| --- | --- |
| random(size=None)  Return random floats in the half-open interval [0.0, 1.0). | source code |

|  |  |
| --- | --- |
| random\_integers(low, high=None, size=None)  Return random integers between `low` and `high`, inclusive. | source code |

|  |  |
| --- | --- |
| random\_sample(size=None)  Return random floats in the half-open interval [0.0, 1.0). | source code |

|  |  |
| --- | --- |
| ranf(size=None)  Return random floats in the half-open interval [0.0, 1.0). | source code |

|  |  |
| --- | --- |
| rayleigh(scale=1.0, size=None)  Draw samples from a Rayleigh distribution. | source code |

|  |  |
| --- | --- |
| sample(size=None)  Return random floats in the half-open interval [0.0, 1.0). | source code |

|  |  |
| --- | --- |
| seed(seed=None)  Seed the generator. | source code |

|  |  |
| --- | --- |
| set\_state(state)  Set the internal state of the generator from a tuple. | source code |

|  |  |
| --- | --- |
| shuffle(x)  Modify a sequence in-place by shuffling its contents. | source code |

|  |  |
| --- | --- |
| standard\_cauchy(size=None)  Standard Cauchy distribution with mode = 0. | source code |

|  |  |
| --- | --- |
| standard\_exponential(size=None)  Draw samples from the standard exponential distribution. | source code |

|  |  |
| --- | --- |
| standard\_gamma(shape, size=None)  Draw samples from a Standard Gamma distribution. | source code |

|  |  |
| --- | --- |
| standard\_normal(size=None)  Returns samples from a Standard Normal distribution (mean=0, stdev=1). | source code |

|  |  |
| --- | --- |
| standard\_t(df, size=None)  Standard Student's t distribution with df degrees of freedom. | source code |

|  |  |
| --- | --- |
| triangular(left, mode, right, size=None)  Draw samples from the triangular distribution. | source code |

|  |  |
| --- | --- |
| uniform(low=0.0, high=1.0, size=1)  Draw samples from a uniform distribution. | source code |

|  |  |
| --- | --- |
| vonmises(mu=0.0, kappa=1.0, size=None)  Draw samples from a von Mises distribution. | source code |

|  |  |
| --- | --- |
| wald(mean, scale, size=None)  Draw samples from a Wald, or Inverse Gaussian, distribution. | source code |

|  |  |
| --- | --- |
| weibull(a, size=None)  Weibull distribution. | source code |

|  |  |
| --- | --- |
| zipf(a, size=None)  Draw samples from a Zipf distribution. | source code |


|  |  |
| --- | --- |
| Variables | [hide private] |

ALLOW\_THREADS = `1`

BUFSIZE = `10000`

CLIP = `0`

DAILY = `3`

ERR\_CALL = `3`

ERR\_DEFAULT = `0`

ERR\_DEFAULT2 = `2084`

ERR\_IGNORE = `0`

ERR\_LOG = `5`

ERR\_PRINT = `4`

ERR\_RAISE = `2`

ERR\_WARN = `1`

FLOATING\_POINT\_SUPPORT = `1`

FPE\_DIVIDEBYZERO = `1`

FPE\_INVALID = `8`

FPE\_OVERFLOW = `2`

FPE\_UNDERFLOW = `4`

FR = `FR`

False\_ = `False`

HOURLY = `4`

Inf = `inf`

Infinity = `inf`

MAXDIMS = `32`

MINUTELY = `5`

MO = `MO`

MONTHLY = `1`

NAN = `nan`

NINF = `-inf`

NZERO = `-0.0`

NaN = `nan`

PINF = `inf`

PZERO = `0.0`

RAISE = `2`

SA = `SA`

SECONDLY = `6`

SHIFT\_DIVIDEBYZERO = `0`

SHIFT\_INVALID = `9`

SHIFT\_OVERFLOW = `3`

SHIFT\_UNDERFLOW = `6`

SU = `SU`

ScalarType = `(<type 'int'>, <type 'float'>, <type 'complex'>, ...`

TH = `TH`

TU = `TU`

True\_ = `True`

UFUNC\_BUFSIZE\_DEFAULT = `10000`

UFUNC\_PYVALS\_NAME = `'UFUNC_PYVALS'`

WE = `WE`

WEEKLY = `2`

WRAP = `1`

YEARLY = `0`

\_classes = `['ContClass']`

\_constants = `['curve_list', 'curve_args_list', 'auto_list']`

\_pydstool\_path = `'/Users/robclewley/PyDSTool'`

absolute = `<ufunc 'absolute'>`

add = `<ufunc 'add'>`

arccos = `<ufunc 'arccos'>`

arccosh = `<ufunc 'arccosh'>`

arcsin = `<ufunc 'arcsin'>`

arcsinh = `<ufunc 'arcsinh'>`

arctan = `<ufunc 'arctan'>`

arctan2 = `<ufunc 'arctan2'>`

arctanh = `<ufunc 'arctanh'>`

auto\_list = `['LC-C']`

bitwise\_and = `<ufunc 'bitwise_and'>`

bitwise\_not = `<ufunc 'invert'>`

bitwise\_or = `<ufunc 'bitwise_or'>`

bitwise\_xor = `<ufunc 'bitwise_xor'>`

c\_ = `<numpy.lib.index_tricks.CClass object at 0x1154330>`

cast = `{<type 'numpy.int64'>: <function <lambda> at 0x10a8bb0>...`

ceil = `<ufunc 'ceil'>`

colorbar\_doc = `'\n\nAdd a colorbar to a plot.\n\nFunction sign...`

conj = `<ufunc 'conjugate'>`

conjugate = `<ufunc 'conjugate'>`

copysign = `<ufunc 'copysign'>`

cos = `<ufunc 'cos'>`

cosh = `<ufunc 'cosh'>`

curve\_args\_list = `['verbosity']`

curve\_list = `{'EP-C': <class 'PyDSTool.PyCont.Continuation.Equ...`

deg2rad = `<ufunc 'deg2rad'>`

degrees = `<ufunc 'degrees'>`

divide = `<ufunc 'divide'>`

e = `2.71828182846`

equal = `<ufunc 'equal'>`

exp = `<ufunc 'exp'>`

exp2 = `<ufunc 'exp2'>`

expm1 = `<ufunc 'expm1'>`

fabs = `<ufunc 'fabs'>`

floor = `<ufunc 'floor'>`

floor\_divide = `<ufunc 'floor_divide'>`

fmax = `<ufunc 'fmax'>`

fmin = `<ufunc 'fmin'>`

fmod = `<ufunc 'fmod'>`

frexp = `<ufunc 'frexp'>`

greater = `<ufunc 'greater'>`

greater\_equal = `<ufunc 'greater_equal'>`

hypot = `<ufunc 'hypot'>`

index\_exp = `<numpy.lib.index_tricks.IndexExpression object at ...`

inf = `inf`

infty = `inf`

invert = `<ufunc 'invert'>`

isfinite = `<ufunc 'isfinite'>`

isinf = `<ufunc 'isinf'>`

isnan = `<ufunc 'isnan'>`

ldexp = `<ufunc 'ldexp'>`

left\_shift = `<ufunc 'left_shift'>`

less = `<ufunc 'less'>`

less\_equal = `<ufunc 'less_equal'>`

little\_endian = `True`

log = `<ufunc 'log'>`

log10 = `<ufunc 'log10'>`

log1p = `<ufunc 'log1p'>`

logaddexp = `<ufunc 'logaddexp'>`

logaddexp2 = `<ufunc 'logaddexp2'>`

logical\_and = `<ufunc 'logical_and'>`

logical\_not = `<ufunc 'logical_not'>`

logical\_or = `<ufunc 'logical_or'>`

logical\_xor = `<ufunc 'logical_xor'>`

maximum = `<ufunc 'maximum'>`

mgrid = `<numpy.lib.index_tricks.nd_grid object at 0x113d490>`

minimum = `<ufunc 'minimum'>`

mod = `<ufunc 'remainder'>`

modf = `<ufunc 'modf'>`

multiply = `<ufunc 'multiply'>`

nan = `nan`

nbytes = `{<type 'numpy.int64'>: 8, <type 'numpy.int16'>: 2, <t...`

negative = `<ufunc 'negative'>`

newaxis = `None`

nextafter = `<ufunc 'nextafter'>`

not\_equal = `<ufunc 'not_equal'>`

ogrid = `<numpy.lib.index_tricks.nd_grid object at 0x113dbb0>`

ones\_like = `<ufunc 'ones_like'>`

pi = `3.14159265359`

r\_ = `<numpy.lib.index_tricks.RClass object at 0x11542f0>`

rad2deg = `<ufunc 'rad2deg'>`

radians = `<ufunc 'radians'>`

rcParams = `{'figure.subplot.right': 0.90000000000000002, 'math...`

rcParamsDefault = `{'figure.subplot.right': 0.90000000000000002...`

reciprocal = `<ufunc 'reciprocal'>`

remainder = `<ufunc 'remainder'>`

rerr = `<PyDSTool.Redirector.Redirector object at 0x64d4430>`

right\_shift = `<ufunc 'right_shift'>`

rint = `<ufunc 'rint'>`

rout = `<PyDSTool.Redirector.Redirector object at 0x64dc050>`

s\_ = `<numpy.lib.index_tricks.IndexExpression object at 0x11543f0>`

sctypeDict = `{0: <type 'numpy.bool_'>, 1: <type 'numpy.int8'>,...`

sctypeNA = `{'?': 'Bool', 'B': 'UInt8', 'Bool': <type 'numpy.bo...`

sctypes = `{'complex': [<type 'numpy.complex64'>, <type 'numpy....`

sign = `<ufunc 'sign'>`

signbit = `<ufunc 'signbit'>`

sin = `<ufunc 'sin'>`

sinh = `<ufunc 'sinh'>`

spacing = `<ufunc 'spacing'>`

sqrt = `<ufunc 'sqrt'>`

square = `<ufunc 'square'>`

subtract = `<ufunc 'subtract'>`

tan = `<ufunc 'tan'>`

tanh = `<ufunc 'tanh'>`

true\_divide = `<ufunc 'true_divide'>`

trunc = `<ufunc 'trunc'>`

typeDict = `{0: <type 'numpy.bool_'>, 1: <type 'numpy.int8'>, 2...`

typeNA = `{'?': 'Bool', 'B': 'UInt8', 'Bool': <type 'numpy.bool...`

typecodes = `{'All': '?bhilqpBHILQPfdgFDGSUVOMm', 'AllFloat': '...`


|  |  |
| --- | --- |
| Function Details | [hide private] |

|  |  |
| --- | --- |
| beta(a, b, size=None) | source code |

```
The Beta distribution over ``[0, 1]``.

The Beta distribution is a special case of the Dirichlet distribution,
and is related to the Gamma distribution.  It has the probability
distribution function

.. math:: f(x; a,b) = \frac{1}{B(\alpha, \beta)} x^{\alpha - 1}
                                                 (1 - x)^{\beta - 1},

where the normalisation, B, is the beta function,

.. math:: B(\alpha, \beta) = \int_0^1 t^{\alpha - 1}
                             (1 - t)^{\beta - 1} dt.

It is often seen in Bayesian inference and order statistics.

Parameters
----------
a : float
    Alpha, non-negative.
b : float
    Beta, non-negative.
size : tuple of ints, optional
    The number of samples to draw.  The ouput is packed according to
    the size given.

Returns
-------
out : ndarray
    Array of the given shape, containing values drawn from a
    Beta distribution.
```

|  |  |
| --- | --- |
| binomial(n, p, size=None) | source code |

```
Draw samples from a binomial distribution.

Samples are drawn from a Binomial distribution with specified
parameters, n trials and p probability of success where
n an integer > 0 and p is in the interval [0,1]. (n may be
input as a float, but it is truncated to an integer in use)

Parameters
----------
n : float (but truncated to an integer)
        parameter, > 0.
p : float
        parameter, >= 0 and <=1.
size : {tuple, int}
    Output shape.  If the given shape is, e.g., ``(m, n, k)``, then
    ``m * n * k`` samples are drawn.

Returns
-------
samples : {ndarray, scalar}
          where the values are all integers in  [0, n].

See Also
--------
scipy.stats.distributions.binom : probability density function,
    distribution or cumulative density function, etc.

Notes
-----
The probability density for the Binomial distribution is

.. math:: P(N) = \binom{n}{N}p^N(1-p)^{n-N},

where :math:`n` is the number of trials, :math:`p` is the probability
of success, and :math:`N` is the number of successes.

When estimating the standard error of a proportion in a population by
using a random sample, the normal distribution works well unless the
product p*n <=5, where p = population proportion estimate, and n =
number of samples, in which case the binomial distribution is used
instead. For example, a sample of 15 people shows 4 who are left
handed, and 11 who are right handed. Then p = 4/15 = 27%. 0.27*15 = 4,
so the binomial distribution should be used in this case.

References
----------
.. [1] Dalgaard, Peter, "Introductory Statistics with R",
       Springer-Verlag, 2002.
.. [2] Glantz, Stanton A. "Primer of Biostatistics.", McGraw-Hill,
       Fifth Edition, 2002.
.. [3] Lentner, Marvin, "Elementary Applied Statistics", Bogden
       and Quigley, 1972.
.. [4] Weisstein, Eric W. "Binomial Distribution." From MathWorld--A
       Wolfram Web Resource.
       http://mathworld.wolfram.com/BinomialDistribution.html
.. [5] Wikipedia, "Binomial-distribution",
       http://en.wikipedia.org/wiki/Binomial_distribution

Examples
--------
Draw samples from the distribution:

>>> n, p = 10, .5 # number of trials, probability of each trial
>>> s = np.random.binomial(n, p, 1000)
# result of flipping a coin 10 times, tested 1000 times.

A real world example. A company drills 9 wild-cat oil exploration
wells, each with an estimated probability of success of 0.1. All nine
wells fail. What is the probability of that happening?

Let's do 20,000 trials of the model, and count the number that
generate zero positive results.

>>> sum(np.random.binomial(9,0.1,20000)==0)/20000.
answer = 0.38885, or 38%.
```

|  |  |
| --- | --- |
| bytes(length) | source code |

```
Return random bytes.

Parameters
----------
length : int
    Number of random bytes.

Returns
-------
out : str
    String of length `N`.

Examples
--------
>>> np.random.bytes(10)
' eh\x85\x022SZ\xbf\xa4' #random
```

|  |  |
| --- | --- |
| chisquare(df, size=None) | source code |

```
Draw samples from a chi-square distribution.

When `df` independent random variables, each with standard
normal distributions (mean 0, variance 1), are squared and summed,
the resulting distribution is chi-square (see Notes).  This
distribution is often used in hypothesis testing.

Parameters
----------
df : int
     Number of degrees of freedom.
size : tuple of ints, int, optional
     Size of the returned array.  By default, a scalar is
     returned.

Returns
-------
output : ndarray
    Samples drawn from the distribution, packed in a `size`-shaped
    array.

Raises
------
ValueError
    When `df` <= 0 or when an inappropriate `size` (e.g. ``size=-1``)
    is given.

Notes
-----
The variable obtained by summing the squares of `df` independent,
standard normally distributed random variables:

.. math:: Q = \sum_{i=0}^{\mathtt{df}} X^2_i

is chi-square distributed, denoted

.. math:: Q \sim \chi^2_k.

The probability density function of the chi-squared distribution is

.. math:: p(x) = \frac{(1/2)^{k/2}}{\Gamma(k/2)}
                 x^{k/2 - 1} e^{-x/2},

where :math:`\Gamma` is the gamma function,

.. math:: \Gamma(x) = \int_0^{-\infty} t^{x - 1} e^{-t} dt.

References
----------
.. [1] NIST/SEMATECH e-Handbook of Statistical Methods,
       http://www.itl.nist.gov/div898/handbook/eda/section3/eda3666.htm
.. [2] Wikipedia, "Chi-square distribution",
       http://en.wikipedia.org/wiki/Chi-square_distribution

Examples
--------
>>> np.random.chisquare(2,4)
array([ 1.89920014,  9.00867716,  3.13710533,  5.62318272])
```

|  |  |
| --- | --- |
| exponential(scale=1.0, size=None) | source code |

```
Exponential distribution.

Its probability density function is

.. math:: f(x; \frac{1}{\beta}) = \frac{1}{\beta} \exp(-\frac{x}{\beta}),

for ``x > 0`` and 0 elsewhere. :math:`\beta` is the scale parameter,
which is the inverse of the rate parameter :math:`\lambda = 1/\beta`.
The rate parameter is an alternative, widely used parameterization
of the exponential distribution [3]_.

The exponential distribution is a continuous analogue of the
geometric distribution.  It describes many common situations, such as
the size of raindrops measured over many rainstorms [1]_, or the time
between page requests to Wikipedia [2]_.

Parameters
----------
scale : float
    The scale parameter, :math:`\beta = 1/\lambda`.
size : tuple of ints
    Number of samples to draw.  The output is shaped
    according to `size`.

References
----------
.. [1] Peyton Z. Peebles Jr., "Probability, Random Variables and
       Random Signal Principles", 4th ed, 2001, p. 57.
.. [2] "Poisson Process", Wikipedia,
       http://en.wikipedia.org/wiki/Poisson_process
.. [3] "Exponential Distribution, Wikipedia,
       http://en.wikipedia.org/wiki/Exponential_distribution
```

|  |  |
| --- | --- |
| f(dfnum, dfden, size=None) | source code |

```
Draw samples from a F distribution.

Samples are drawn from an F distribution with specified parameters,
`dfnum` (degrees of freedom in numerator) and `dfden` (degrees of freedom
in denominator), where both parameters should be greater than zero.

The random variate of the F distribution (also known as the
Fisher distribution) is a continuous probability distribution
that arises in ANOVA tests, and is the ratio of two chi-square
variates.

Parameters
----------
dfnum : float
    Degrees of freedom in numerator. Should be greater than zero.
dfden : float
    Degrees of freedom in denominator. Should be greater than zero.
size : {tuple, int}, optional
    Output shape.  If the given shape is, e.g., ``(m, n, k)``,
    then ``m * n * k`` samples are drawn. By default only one sample
    is returned.

Returns
-------
samples : {ndarray, scalar}
    Samples from the Fisher distribution.

See Also
--------
scipy.stats.distributions.f : probability density function,
    distribution or cumulative density function, etc.

Notes
-----

The F statistic is used to compare in-group variances to between-group
variances. Calculating the distribution depends on the sampling, and
so it is a function of the respective degrees of freedom in the
problem.  The variable `dfnum` is the number of samples minus one, the
between-groups degrees of freedom, while `dfden` is the within-groups
degrees of freedom, the sum of the number of samples in each group
minus the number of groups.

References
----------
.. [1] Glantz, Stanton A. "Primer of Biostatistics.", McGraw-Hill,
       Fifth Edition, 2002.
.. [2] Wikipedia, "F-distribution",
       http://en.wikipedia.org/wiki/F-distribution

Examples
--------
An example from Glantz[1], pp 47-40.
Two groups, children of diabetics (25 people) and children from people
without diabetes (25 controls). Fasting blood glucose was measured,
case group had a mean value of 86.1, controls had a mean value of
82.2. Standard deviations were 2.09 and 2.49 respectively. Are these
data consistent with the null hypothesis that the parents diabetic
status does not affect their children's blood glucose levels?
Calculating the F statistic from the data gives a value of 36.01.

Draw samples from the distribution:

>>> dfnum = 1. # between group degrees of freedom
>>> dfden = 48. # within groups degrees of freedom
>>> s = np.random.f(dfnum, dfden, 1000)

The lower bound for the top 1% of the samples is :

>>> sort(s)[-10]
7.61988120985

So there is about a 1% chance that the F statistic will exceed 7.62,
the measured value is 36, so the null hypothesis is rejected at the 1%
level.
```

|  |  |
| --- | --- |
| gamma(shape, scale=1.0, size=None) | source code |

```
Draw samples from a Gamma distribution.

Samples are drawn from a Gamma distribution with specified parameters,
`shape` (sometimes designated "k") and `scale` (sometimes designated
"theta"), where both parameters are > 0.

Parameters
----------
shape : scalar > 0
    The shape of the gamma distribution.
scale : scalar > 0, optional
    The scale of the gamma distribution.  Default is equal to 1.
size : shape_tuple, optional
    Output shape.  If the given shape is, e.g., ``(m, n, k)``, then
    ``m * n * k`` samples are drawn.

Returns
-------
out : ndarray, float
    Returns one sample unless `size` parameter is specified.

See Also
--------
scipy.stats.distributions.gamma : probability density function,
    distribution or cumulative density function, etc.

Notes
-----
The probability density for the Gamma distribution is

.. math:: p(x) = x^{k-1}\frac{e^{-x/\theta}}{\theta^k\Gamma(k)},

where :math:`k` is the shape and :math:`\theta` the scale,
and :math:`\Gamma` is the Gamma function.

The Gamma distribution is often used to model the times to failure of
electronic components, and arises naturally in processes for which the
waiting times between Poisson distributed events are relevant.

References
----------
.. [1] Weisstein, Eric W. "Gamma Distribution." From MathWorld--A
       Wolfram Web Resource.
       http://mathworld.wolfram.com/GammaDistribution.html
.. [2] Wikipedia, "Gamma-distribution",
       http://en.wikipedia.org/wiki/Gamma-distribution

Examples
--------
Draw samples from the distribution:

>>> shape, scale = 2., 2. # mean and dispersion
>>> s = np.random.gamma(shape, scale, 1000)

Display the histogram of the samples, along with
the probability density function:

>>> import matplotlib.pyplot as plt
>>> import scipy.special as sps
>>> count, bins, ignored = plt.hist(s, 50, normed=True)
>>> y = bins**(shape-1)*(np.exp(-bins/scale) /
...                      (sps.gamma(shape)*scale**shape))
>>> plt.plot(bins, y, linewidth=2, color='r')
>>> plt.show()
```

|  |  |
| --- | --- |
| geometric(p, size=None) | source code |

```
Draw samples from the geometric distribution.

Bernoulli trials are experiments with one of two outcomes:
success or failure (an example of such an experiment is flipping
a coin).  The geometric distribution models the number of trials
that must be run in order to achieve success.  It is therefore
supported on the positive integers, ``k = 1, 2, ...``.

The probability mass function of the geometric distribution is

.. math:: f(k) = (1 - p)^{k - 1} p

where `p` is the probability of success of an individual trial.

Parameters
----------
p : float
    The probability of success of an individual trial.
size : tuple of ints
    Number of values to draw from the distribution.  The output
    is shaped according to `size`.

Returns
-------
out : ndarray
    Samples from the geometric distribution, shaped according to
    `size`.

Examples
--------
Draw ten thousand values from the geometric distribution,
with the probability of an individual success equal to 0.35:

>>> z = np.random.geometric(p=0.35, size=10000)

How many trials succeeded after a single run?

>>> (z == 1).sum() / 10000.
0.34889999999999999 #random
```

|  |  |
| --- | --- |
| get\_state() | source code |

```
Return a tuple representing the internal state of the generator.

For more details, see `set_state`.

Returns
-------
out : tuple(str, ndarray of 624 uints, int, int, float)
    The returned tuple has the following items:

    1. the string 'MT19937'.
    2. a 1-D array of 624 unsigned integer keys.
    3. an integer ``pos``.
    4. an integer ``has_gauss``.
    5. a float ``cached_gaussian``.

See Also
--------
set_state

Notes
-----
`set_state` and `get_state` are not needed to work with any of the
random distributions in NumPy. If the internal state is manually altered,
the user should know exactly what he/she is doing.
```

|  |  |
| --- | --- |
| gumbel(loc=0.0, scale=1.0, size=None) | source code |

```
Gumbel distribution.

Draw samples from a Gumbel distribution with specified location (or mean)
and scale (or standard deviation).

The Gumbel (or Smallest Extreme Value (SEV) or the Smallest Extreme Value
Type I) distribution is one of a class of Generalized Extreme Value (GEV)
distributions used in modeling extreme value problems.  The Gumbel is a
special case of the Extreme Value Type I distribution for maximums from
distributions with "exponential-like" tails, it may be derived by
considering a Gaussian process of measurements, and generating the pdf for
the maximum values from that set of measurements (see examples).

Parameters
----------
loc : float
    The location of the mode of the distribution.
scale : float
    The scale parameter of the distribution.
size : tuple of ints
    Output shape.  If the given shape is, e.g., ``(m, n, k)``, then
    ``m * n * k`` samples are drawn.

See Also
--------
scipy.stats.gumbel : probability density function,
    distribution or cumulative density function, etc.
weibull, scipy.stats.genextreme

Notes
-----
The probability density for the Gumbel distribution is

.. math:: p(x) = \frac{e^{-(x - \mu)/ \beta}}{\beta} e^{ -e^{-(x - \mu)/
          \beta}},

where :math:`\mu` is the mode, a location parameter, and :math:`\beta`
is the scale parameter.

The Gumbel (named for German mathematician Emil Julius Gumbel) was used
very early in the hydrology literature, for modeling the occurrence of
flood events. It is also used for modeling maximum wind speed and rainfall
rates.  It is a "fat-tailed" distribution - the probability of an event in
the tail of the distribution is larger than if one used a Gaussian, hence
the surprisingly frequent occurrence of 100-year floods. Floods were
initially modeled as a Gaussian process, which underestimated the frequency
of extreme events.

It is one of a class of extreme value distributions, the Generalized
Extreme Value (GEV) distributions, which also includes the Weibull and
Frechet.

The function has a mean of :math:`\mu + 0.57721\beta` and a variance of
:math:`\frac{\pi^2}{6}\beta^2`.

References
----------
.. [1] Gumbel, E.J. (1958). Statistics of Extremes. Columbia University
       Press.
.. [2] Reiss, R.-D. and Thomas M. (2001), Statistical Analysis of Extreme
       Values, from Insurance, Finance, Hydrology and Other Fields,
       Birkhauser Verlag, Basel: Boston : Berlin.
.. [3] Wikipedia, "Gumbel distribution",
       http://en.wikipedia.org/wiki/Gumbel_distribution

Examples
--------
Draw samples from the distribution:

>>> mu, beta = 0, 0.1 # location and scale
>>> s = np.random.gumbel(mu, beta, 1000)

Display the histogram of the samples, along with
the probability density function:

>>> import matplotlib.pyplot as plt
>>> count, bins, ignored = plt.hist(s, 30, normed=True)
>>> plt.plot(bins, (1/beta)*np.exp(-(bins - mu)/beta)
...          * np.exp( -np.exp( -(bins - mu) /beta) ),
...          linewidth=2, color='r')
>>> plt.show()

Show how an extreme value distribution can arise from a Gaussian process
and compare to a Gaussian:

>>> means = []
>>> maxima = []
>>> for i in range(0,1000) :
...    a = np.random.normal(mu, beta, 1000)
...    means.append(a.mean())
...    maxima.append(a.max())
>>> count, bins, ignored = plt.hist(maxima, 30, normed=True)
>>> beta = np.std(maxima)*np.pi/np.sqrt(6)
>>> mu = np.mean(maxima) - 0.57721*beta
>>> plt.plot(bins, (1/beta)*np.exp(-(bins - mu)/beta)
...          * np.exp(-np.exp(-(bins - mu)/beta)),
...          linewidth=2, color='r')
>>> plt.plot(bins, 1/(beta * np.sqrt(2 * np.pi))
...          * np.exp(-(bins - mu)**2 / (2 * beta**2)),
...          linewidth=2, color='g')
>>> plt.show()
```

|  |  |
| --- | --- |
| hypergeometric(ngood, nbad, nsample, size=None) | source code |

```
Draw samples from a Hypergeometric distribution.

Samples are drawn from a Hypergeometric distribution with specified
parameters, ngood (ways to make a good selection), nbad (ways to make
a bad selection), and nsample = number of items sampled, which is less
than or equal to the sum ngood + nbad.

Parameters
----------
ngood : float (but truncated to an integer)
        parameter, > 0.
nbad  : float
        parameter, >= 0.
nsample  : float
           parameter, > 0 and <= ngood+nbad
size : {tuple, int}
    Output shape.  If the given shape is, e.g., ``(m, n, k)``, then
    ``m * n * k`` samples are drawn.

Returns
-------
samples : {ndarray, scalar}
          where the values are all integers in  [0, n].

See Also
--------
scipy.stats.distributions.hypergeom : probability density function,
    distribution or cumulative density function, etc.

Notes
-----
The probability density for the Hypergeometric distribution is

.. math:: P(x) = \frac{\binom{m}{n}\binom{N-m}{n-x}}{\binom{N}{n}},

where :math:`0 \le x \le m` and :math:`n+m-N \le x \le n`

for P(x) the probability of x successes, n = ngood, m = nbad, and
N = number of samples.

Consider an urn with black and white marbles in it, ngood of them
black and nbad are white. If you draw nsample balls without
replacement, then the Hypergeometric distribution describes the
distribution of black balls in the drawn sample.

Note that this distribution is very similar to the Binomial
distribution, except that in this case, samples are drawn without
replacement, whereas in the Binomial case samples are drawn with
replacement (or the sample space is infinite). As the sample space
becomes large, this distribution approaches the Binomial.

References
----------
.. [1] Lentner, Marvin, "Elementary Applied Statistics", Bogden
       and Quigley, 1972.
.. [2] Weisstein, Eric W. "Hypergeometric Distribution." From
       MathWorld--A Wolfram Web Resource.
       http://mathworld.wolfram.com/HypergeometricDistribution.html
.. [3] Wikipedia, "Hypergeometric-distribution",
       http://en.wikipedia.org/wiki/Hypergeometric-distribution

Examples
--------
Draw samples from the distribution:

>>> ngood, nbad, nsamp = 100, 2, 10
# number of good, number of bad, and number of samples
>>> s = np.random.hypergeometric(ngood, nbad, nsamp, 1000)
>>> hist(s)
#   note that it is very unlikely to grab both bad items

Suppose you have an urn with 15 white and 15 black marbles.
If you pull 15 marbles at random, how likely is it that
12 or more of them are one color?

>>> s = np.random.hypergeometric(15, 15, 15, 100000)
>>> sum(s>=12)/100000. + sum(s<=3)/100000.
#   answer = 0.003 ... pretty unlikely!
```

|  |  |
| --- | --- |
| laplace(loc=0.0, scale=1.0, size=None) | source code |

```
Draw samples from the Laplace or double exponential distribution with
specified location (or mean) and scale (decay).

The Laplace distribution is similar to the Gaussian/normal distribution,
but is sharper at the peak and has fatter tails. It represents the
difference between two independent, identically distributed exponential
random variables.

Parameters
----------
loc : float
    The position, :math:`\mu`, of the distribution peak.
scale : float
    :math:`\lambda`, the exponential decay.

Notes
-----
It has the probability density function

.. math:: f(x; \mu, \lambda) = \frac{1}{2\lambda}
                               \exp\left(-\frac{|x - \mu|}{\lambda}\right).

The first law of Laplace, from 1774, states that the frequency of an error
can be expressed as an exponential function of the absolute magnitude of
the error, which leads to the Laplace distribution. For many problems in
Economics and Health sciences, this distribution seems to model the data
better than the standard Gaussian distribution


References
----------
.. [1] Abramowitz, M. and Stegun, I. A. (Eds.). Handbook of Mathematical
       Functions with Formulas, Graphs, and Mathematical Tables, 9th
       printing.  New York: Dover, 1972.

.. [2] The Laplace distribution and generalizations
       By Samuel Kotz, Tomasz J. Kozubowski, Krzysztof Podgorski,
       Birkhauser, 2001.

.. [3] Weisstein, Eric W. "Laplace Distribution."
       From MathWorld--A Wolfram Web Resource.
       http://mathworld.wolfram.com/LaplaceDistribution.html

.. [4] Wikipedia, "Laplace distribution",
       http://en.wikipedia.org/wiki/Laplace_distribution

Examples
--------
Draw samples from the distribution

>>> loc, scale = 0., 1.
>>> s = np.random.laplace(loc, scale, 1000)

Display the histogram of the samples, along with
the probability density function:

>>> import matplotlib.pyplot as plt
>>> count, bins, ignored = plt.hist(s, 30, normed=True)
>>> x = np.arange(-8., 8., .01)
>>> pdf = np.exp(-abs(x-loc/scale))/(2.*scale)
>>> plt.plot(x, pdf)

Plot Gaussian for comparison:

>>> g = (1/(scale * np.sqrt(2 * np.pi)) * 
...      np.exp( - (x - loc)**2 / (2 * scale**2) ))
>>> plt.plot(x,g)
```

|  |  |
| --- | --- |
| logistic(loc=0.0, scale=1.0, size=None) | source code |

```
Draw samples from a Logistic distribution.

Samples are drawn from a Logistic distribution with specified
parameters, loc (location or mean, also median), and scale (>0).

Parameters
----------
loc : float

scale : float > 0.

size : {tuple, int}
    Output shape.  If the given shape is, e.g., ``(m, n, k)``, then
    ``m * n * k`` samples are drawn.

Returns
-------
samples : {ndarray, scalar}
          where the values are all integers in  [0, n].

See Also
--------
scipy.stats.distributions.logistic : probability density function,
    distribution or cumulative density function, etc.

Notes
-----
The probability density for the Logistic distribution is

.. math:: P(x) = P(x) = \frac{e^{-(x-\mu)/s}}{s(1+e^{-(x-\mu)/s})^2},

where :math:`\mu` = location and :math:`s` = scale.

The Logistic distribution is used in Extreme Value problems where it
can act as a mixture of Gumbel distributions, in Epidemiology, and by
the World Chess Federation (FIDE) where it is used in the Elo ranking
system, assuming the performance of each player is a logistically
distributed random variable.

References
----------
.. [1] Reiss, R.-D. and Thomas M. (2001), Statistical Analysis of Extreme
       Values, from Insurance, Finance, Hydrology and Other Fields,
       Birkhauser Verlag, Basel, pp 132-133.
.. [2] Weisstein, Eric W. "Logistic Distribution." From
       MathWorld--A Wolfram Web Resource.
       http://mathworld.wolfram.com/LogisticDistribution.html
.. [3] Wikipedia, "Logistic-distribution",
       http://en.wikipedia.org/wiki/Logistic-distribution

Examples
--------
Draw samples from the distribution:

>>> loc, scale = 10, 1
>>> s = np.random.logistic(loc, scale, 10000)
>>> count, bins, ignored = plt.hist(s, bins=50)

#   plot against distribution

>>> def logist(x, loc, scale):
...     return exp((loc-x)/scale)/(scale*(1+exp((loc-x)/scale))**2)
>>> plt.plot(bins, logist(bins, loc, scale)*count.max()/\
... logist(bins, loc, scale).max())
>>> plt.show()
```

|  |  |
| --- | --- |
| lognormal(mean=0.0, sigma=1.0, size=None) | source code |

```
Return samples drawn from a log-normal distribution.

Draw samples from a log-normal distribution with specified mean, standard
deviation, and shape. Note that the mean and standard deviation are not the
values for the distribution itself, but of the underlying normal
distribution it is derived from.


Parameters
----------
mean : float
    Mean value of the underlying normal distribution
sigma : float, >0.
    Standard deviation of the underlying normal distribution
size : tuple of ints
    Output shape.  If the given shape is, e.g., ``(m, n, k)``, then
    ``m * n * k`` samples are drawn.

See Also
--------
scipy.stats.lognorm : probability density function, distribution,
    cumulative density function, etc.

Notes
-----
A variable `x` has a log-normal distribution if `log(x)` is normally
distributed.

The probability density function for the log-normal distribution is

.. math:: p(x) = \frac{1}{\sigma x \sqrt{2\pi}}
                 e^{(-\frac{(ln(x)-\mu)^2}{2\sigma^2})}

where :math:`\mu` is the mean and :math:`\sigma` is the standard deviation
of the normally distributed logarithm of the variable.

A log-normal distribution results if a random variable is the *product* of
a large number of independent, identically-distributed variables in the
same way that a normal distribution results if the variable is the *sum*
of a large number of independent, identically-distributed variables
(see the last example). It is one of the so-called "fat-tailed"
distributions.

The log-normal distribution is commonly used to model the lifespan of units
with fatigue-stress failure modes. Since this includes
most mechanical systems, the log-normal distribution has widespread
application.

It is also commonly used to model oil field sizes, species abundance, and
latent periods of infectious diseases.

References
----------
.. [1] Eckhard Limpert, Werner A. Stahel, and Markus Abbt, "Log-normal
       Distributions across the Sciences: Keys and Clues", May 2001
       Vol. 51 No. 5 BioScience
       http://stat.ethz.ch/~stahel/lognormal/bioscience.pdf
.. [2] Reiss, R.D., Thomas, M.(2001), Statistical Analysis of Extreme
       Values, Birkhauser Verlag, Basel, pp 31-32.
.. [3] Wikipedia, "Lognormal distribution",
       http://en.wikipedia.org/wiki/Lognormal_distribution

Examples
--------
Draw samples from the distribution:

>>> mu, sigma = 3., 1. # mean and standard deviation
>>> s = np.random.lognormal(mu, sigma, 1000)

Display the histogram of the samples, along with
the probability density function:

>>> import matplotlib.pyplot as plt
>>> count, bins, ignored = plt.hist(s, 100, normed=True, align='mid')

>>> x = np.linspace(min(bins), max(bins), 10000)
>>> pdf = (np.exp(-(np.log(x) - mu)**2 / (2 * sigma**2))
...        / (x * sigma * np.sqrt(2 * np.pi)))

>>> plt.plot(x, pdf, linewidth=2, color='r')
>>> plt.axis('tight')
>>> plt.show()

Demonstrate that taking the products of random samples from a uniform
distribution can be fit well by a log-normal probability density function.

>>> # Generate a thousand samples: each is the product of 100 random
>>> # values, drawn from a normal distribution.
>>> b = []
>>> for i in range(1000):
...    a = 10. + np.random.random(100)
...    b.append(np.product(a))

>>> b = np.array(b) / np.min(b) # scale values to be positive

>>> count, bins, ignored = plt.hist(b, 100, normed=True, align='center')

>>> sigma = np.std(np.log(b))
>>> mu = np.mean(np.log(b))

>>> x = np.linspace(min(bins), max(bins), 10000)
>>> pdf = (np.exp(-(np.log(x) - mu)**2 / (2 * sigma**2))
...        / (x * sigma * np.sqrt(2 * np.pi)))

>>> plt.plot(x, pdf, color='r', linewidth=2)
>>> plt.show()
```

|  |  |
| --- | --- |
| logseries(p, size=None) | source code |

```
Draw samples from a Logarithmic Series distribution.

Samples are drawn from a Log Series distribution with specified
parameter, p (probability, 0 < p < 1).

Parameters
----------
loc : float

scale : float > 0.

size : {tuple, int}
    Output shape.  If the given shape is, e.g., ``(m, n, k)``, then
    ``m * n * k`` samples are drawn.

Returns
-------
samples : {ndarray, scalar}
          where the values are all integers in  [0, n].

See Also
--------
scipy.stats.distributions.logser : probability density function,
    distribution or cumulative density function, etc.

Notes
-----
The probability density for the Log Series distribution is

.. math:: P(k) = \frac{-p^k}{k \ln(1-p)},

where p = probability.

The Log Series distribution is frequently used to represent species
richness and occurrence, first proposed by Fisher, Corbet, and
Williams in 1943 [2].  It may also be used to model the numbers of
occupants seen in cars [3].

References
----------
.. [1] Buzas, Martin A.; Culver, Stephen J.,  Understanding regional
       species diversity through the log series distribution of
       occurrences: BIODIVERSITY RESEARCH Diversity & Distributions,
       Volume 5, Number 5, September 1999 , pp. 187-195(9).
.. [2] Fisher, R.A,, A.S. Corbet, and C.B. Williams. 1943. The
       relation between the number of species and the number of
       individuals in a random sample of an animal population.
       Journal of Animal Ecology, 12:42-58.
.. [3] D. J. Hand, F. Daly, D. Lunn, E. Ostrowski, A Handbook of Small
       Data Sets, CRC Press, 1994.
.. [4] Wikipedia, "Logarithmic-distribution",
       http://en.wikipedia.org/wiki/Logarithmic-distribution

Examples
--------
Draw samples from the distribution:

>>> a = .6
>>> s = np.random.logseries(a, 10000)
>>> count, bins, ignored = plt.hist(s)

#   plot against distribution

>>> def logseries(k, p):
...     return -p**k/(k*log(1-p))
>>> plt.plot(bins, logseries(bins, a)*count.max()/
             logseries(bins, a).max(), 'r')
>>> plt.show()
```

|  |  |
| --- | --- |
| multinomial(n, pvals, size=None) | source code |

```
Draw samples from a multinomial distribution.

The multinomial distribution is a multivariate generalisation of the
binomial distribution.  Take an experiment with one of ``p``
possible outcomes.  An example of such an experiment is throwing a dice,
where the outcome can be 1 through 6.  Each sample drawn from the
distribution represents `n` such experiments.  Its values,
``X_i = [X_0, X_1, ..., X_p]``, represent the number of times the outcome
was ``i``.

Parameters
----------
n : int
    Number of experiments.
pvals : sequence of floats, length p
    Probabilities of each of the ``p`` different outcomes.  These
    should sum to 1 (however, the last element is always assumed to
    account for the remaining probability, as long as
    ``sum(pvals[:-1]) <= 1)``.
size : tuple of ints
    Given a `size` of ``(M, N, K)``, then ``M*N*K`` samples are drawn,
    and the output shape becomes ``(M, N, K, p)``, since each sample
    has shape ``(p,)``.

Examples
--------
Throw a dice 20 times:

>>> np.random.multinomial(20, [1/6.]*6, size=1)
array([[4, 1, 7, 5, 2, 1]])

It landed 4 times on 1, once on 2, etc.

Now, throw the dice 20 times, and 20 times again:

>>> np.random.multinomial(20, [1/6.]*6, size=2)
array([[3, 4, 3, 3, 4, 3],
       [2, 4, 3, 4, 0, 7]])

For the first run, we threw 3 times 1, 4 times 2, etc.  For the second,
we threw 2 times 1, 4 times 2, etc.

A loaded dice is more likely to land on number 6:

>>> np.random.multinomial(100, [1/7.]*5)
array([13, 16, 13, 16, 42])
```

|  |  |
| --- | --- |
| multivariate\_normal(mean, cov, size=...) | source code |

```
Draw random samples from a multivariate normal distribution.

The multivariate normal, multinormal or Gaussian distribution is a
generalisation of the one-dimensional normal distribution to higher
dimensions.

Such a distribution is specified by its mean and covariance matrix,
which are analogous to the mean (average or "centre") and variance
(standard deviation squared or "width") of the one-dimensional normal
distribution.

Parameters
----------
mean : (N,) ndarray
    Mean of the N-dimensional distribution.
cov : (N,N) ndarray
    Covariance matrix of the distribution.
size : tuple of ints, optional
    Given a shape of, for example, (m,n,k), m*n*k samples are
    generated, and packed in an m-by-n-by-k arrangement.  Because each
    sample is N-dimensional, the output shape is (m,n,k,N).  If no
    shape is specified, a single sample is returned.

Returns
-------
out : ndarray
    The drawn samples, arranged according to `size`.  If the
    shape given is (m,n,...), then the shape of `out` is is
    (m,n,...,N).

    In other words, each entry ``out[i,j,...,:]`` is an N-dimensional
    value drawn from the distribution.

Notes
-----
The mean is a coordinate in N-dimensional space, which represents the
location where samples are most likely to be generated.  This is
analogous to the peak of the bell curve for the one-dimensional or
univariate normal distribution.

Covariance indicates the level to which two variables vary together.
From the multivariate normal distribution, we draw N-dimensional
samples, :math:`X = [x_1, x_2, ... x_N]`.  The covariance matrix
element :math:`C_{ij}` is the covariance of :math:`x_i` and :math:`x_j`.
The element :math:`C_{ii}` is the variance of :math:`x_i` (i.e. its
"spread").

Instead of specifying the full covariance matrix, popular
approximations include:

  - Spherical covariance (`cov` is a multiple of the identity matrix)
  - Diagonal covariance (`cov` has non-negative elements, and only on
    the diagonal)

This geometrical property can be seen in two dimensions by plotting
generated data-points:

>>> mean = [0,0]
>>> cov = [[1,0],[0,100]] # diagonal covariance, points lie on x or y-axis

>>> import matplotlib.pyplot as plt
>>> x,y = np.random.multivariate_normal(mean,cov,5000).T
>>> plt.plot(x,y,'x'); plt.axis('equal'); plt.show()

Note that the covariance matrix must be non-negative definite.

References
----------
.. [1] A. Papoulis, "Probability, Random Variables, and Stochastic
       Processes," 3rd ed., McGraw-Hill Companies, 1991
.. [2] R.O. Duda, P.E. Hart, and D.G. Stork, "Pattern Classification,"
       2nd ed., Wiley, 2001.

Examples
--------
>>> mean = (1,2)
>>> cov = [[1,0],[1,0]]
>>> x = np.random.multivariate_normal(mean,cov,(3,3))
>>> x.shape
(3, 3, 2)

The following is probably true, given that 0.6 is roughly twice the
standard deviation:

>>> print list( (x[0,0,:] - mean) < 0.6 )
[True, True]
```

|  |  |
| --- | --- |
| negative\_binomial(n, p, size=None) | source code |

```
Draw samples from a negative_binomial distribution.

Samples are drawn from a negative_Binomial distribution with specified
parameters, `n` trials and `p` probability of success where `n` is an
integer > 0 and `p` is in the interval [0, 1].

Parameters
----------
n : int
    Parameter, > 0.
p : float
    Parameter, >= 0 and <=1.
size : int or tuple of ints
    Output shape. If the given shape is, e.g., ``(m, n, k)``, then
    ``m * n * k`` samples are drawn.

Returns
-------
samples : int or ndarray of ints
    Drawn samples.

Notes
-----
The probability density for the Negative Binomial distribution is

.. math:: P(N;n,p) = \binom{N+n-1}{n-1}p^{n}(1-p)^{N},

where :math:`n-1` is the number of successes, :math:`p` is the probability
of success, and :math:`N+n-1` is the number of trials.

The negative binomial distribution gives the probability of n-1 successes
and N failures in N+n-1 trials, and success on the (N+n)th trial.

If one throws a die repeatedly until the third time a "1" appears, then the
probability distribution of the number of non-"1"s that appear before the
third "1" is a negative binomial distribution.

References
----------
.. [1] Weisstein, Eric W. "Negative Binomial Distribution." From
       MathWorld--A Wolfram Web Resource.
       http://mathworld.wolfram.com/NegativeBinomialDistribution.html
.. [2] Wikipedia, "Negative binomial distribution",
       http://en.wikipedia.org/wiki/Negative_binomial_distribution

Examples
--------
Draw samples from the distribution:

A real world example. A company drills wild-cat oil exploration wells, each
with an estimated probability of success of 0.1.  What is the probability
of having one success for each successive well, that is what is the
probability of a single success after drilling 5 wells, after 6 wells,
etc.?

>>> s = np.random.negative_binomial(1, 0.1, 100000)
>>> for i in range(1, 11):
...    probability = sum(s<i) / 100000.
...    print i, "wells drilled, probability of one success =", probability
```

|  |  |
| --- | --- |
| noncentral\_chisquare(df, nonc, size=None) | source code |

```
Draw samples from a noncentral chi-square distribution.

The noncentral :math:`\chi^2` distribution is a generalisation of
the :math:`\chi^2` distribution.

Parameters
----------
df : int
    Degrees of freedom, should be >= 1.
nonc : float
    Non-centrality, should be > 0.
size : int or tuple of ints
    Shape of the output.

Notes
-----
The probability density function for the noncentral Chi-square distribution
is

.. math:: P(x;df,nonc) = \sum^{\infty}_{i=0}
                       \frac{e^{-nonc/2}(nonc/2)^{i}}{i!}P_{Y_{df+2i}}(x),

where :math:`Y_{q}` is the Chi-square with q degrees of freedom.

In Delhi (2007), it is noted that the noncentral chi-square is useful in
bombing and coverage problems, the probability of killing the point target
given by the noncentral chi-squared distribution.

References
----------
.. [1] Delhi, M.S. Holla, "On a noncentral chi-square distribution in the
       analysis of weapon systems effectiveness", Metrika, Volume 15,
       Number 1 / December, 1970.
.. [2] Wikipedia, "Noncentral chi-square distribution"
       http://en.wikipedia.org/wiki/Noncentral_chi-square_distribution

Examples
--------
Draw values from the distribution and plot the histogram

>>> import matplotlib.pyplot as plt
>>> values = plt.hist(np.random.noncentral_chisquare(3, 20, 100000),
...                   bins=200, normed=True)
>>> plt.show()

Draw values from a noncentral chisquare with very small noncentrality,
and compare to a chisquare.

>>> plt.figure()
>>> values = plt.hist(np.random.noncentral_chisquare(3, .0000001, 100000),
...                   bins=np.arange(0., 25, .1), normed=True)
>>> values2 = plt.hist(np.random.chisquare(3, 100000),
...                    bins=np.arange(0., 25, .1), normed=True)
>>> plt.plot(values[1][0:-1], values[0]-values2[0], 'ob')
>>> plt.show()

Demonstrate how large values of non-centrality lead to a more symmetric
distribution.

>>> plt.figure()
>>> values = plt.hist(np.random.noncentral_chisquare(3, 20, 100000),
...                   bins=200, normed=True)
>>> plt.show()
```

|  |  |
| --- | --- |
| noncentral\_f(dfnum, dfden, nonc, size=None) | source code |

```
Draw samples from the noncentral F distribution.

Samples are drawn from an F distribution with specified parameters,
`dfnum` (degrees of freedom in numerator) and `dfden` (degrees of
freedom in denominator), where both parameters > 1.
`nonc` is the non-centrality parameter.

Parameters
----------
dfnum : int
    Parameter, should be > 1.
dfden : int
    Parameter, should be > 1.
nonc : float
    Parameter, should be >= 0.
size : int or tuple of ints
    Output shape. If the given shape is, e.g., ``(m, n, k)``, then
    ``m * n * k`` samples are drawn.

Returns
-------
samples : scalar or ndarray
    Drawn samples.

Notes
-----
When calculating the power of an experiment (power = probability of
rejecting the null hypothesis when a specific alternative is true) the
non-central F statistic becomes important.  When the null hypothesis is
true, the F statistic follows a central F distribution. When the null
hypothesis is not true, then it follows a non-central F statistic.

References
----------
Weisstein, Eric W. "Noncentral F-Distribution." From MathWorld--A Wolfram
Web Resource.  http://mathworld.wolfram.com/NoncentralF-Distribution.html

Wikipedia, "Noncentral F distribution",
http://en.wikipedia.org/wiki/Noncentral_F-distribution

Examples
--------
In a study, testing for a specific alternative to the null hypothesis
requires use of the Noncentral F distribution. We need to calculate the
area in the tail of the distribution that exceeds the value of the F
distribution for the null hypothesis.  We'll plot the two probability
distributions for comparison.

>>> dfnum = 3 # between group deg of freedom
>>> dfden = 20 # within groups degrees of freedom
>>> nonc = 3.0
>>> nc_vals = np.random.noncentral_f(dfnum, dfden, nonc, 1000000)
>>> NF = np.histogram(nc_vals, bins=50, normed=True)
>>> c_vals = np.random.f(dfnum, dfden, 1000000)
>>> F = np.histogram(c_vals, bins=50, normed=True)
>>> plt.plot(F[1][1:], F[0])
>>> plt.plot(NF[1][1:], NF[0])
>>> plt.show()
```

|  |  |
| --- | --- |
| normal(loc=0.0, scale=1.0, size=None) | source code |

```
Draw random samples from a normal (Gaussian) distribution.

The probability density function of the normal distribution, first
derived by De Moivre and 200 years later by both Gauss and Laplace
independently [2]_, is often called the bell curve because of
its characteristic shape (see the example below).

The normal distributions occurs often in nature.  For example, it
describes the commonly occurring distribution of samples influenced
by a large number of tiny, random disturbances, each with its own
unique distribution [2]_.

Parameters
----------
loc : float
    Mean ("centre") of the distribution.
scale : float
    Standard deviation (spread or "width") of the distribution.
size : tuple of ints
    Output shape.  If the given shape is, e.g., ``(m, n, k)``, then
    ``m * n * k`` samples are drawn.

See Also
--------
scipy.stats.distributions.norm : probability density function,
    distribution or cumulative density function, etc.

Notes
-----
The probability density for the Gaussian distribution is

.. math:: p(x) = \frac{1}{\sqrt{ 2 \pi \sigma^2 }}
                 e^{ - \frac{ (x - \mu)^2 } {2 \sigma^2} },

where :math:`\mu` is the mean and :math:`\sigma` the standard deviation.
The square of the standard deviation, :math:`\sigma^2`, is called the
variance.

The function has its peak at the mean, and its "spread" increases with
the standard deviation (the function reaches 0.607 times its maximum at
:math:`x + \sigma` and :math:`x - \sigma` [2]_).  This implies that
`numpy.random.normal` is more likely to return samples lying close to the
mean, rather than those far away.

References
----------
.. [1] Wikipedia, "Normal distribution",
       http://en.wikipedia.org/wiki/Normal_distribution
.. [2] P. R. Peebles Jr., "Central Limit Theorem" in "Probability, Random
       Variables and Random Signal Principles", 4th ed., 2001,
       pp. 51, 51, 125.

Examples
--------
Draw samples from the distribution:

>>> mu, sigma = 0, 0.1 # mean and standard deviation
>>> s = np.random.normal(mu, sigma, 1000)

Verify the mean and the variance:

>>> abs(mu - np.mean(s)) < 0.01
True

>>> abs(sigma - np.std(s, ddof=1)) < 0.01
True

Display the histogram of the samples, along with
the probability density function:

>>> import matplotlib.pyplot as plt
>>> count, bins, ignored = plt.hist(s, 30, normed=True)
>>> plt.plot(bins, 1/(sigma * np.sqrt(2 * np.pi)) *
...                np.exp( - (bins - mu)**2 / (2 * sigma**2) ),
...          linewidth=2, color='r')
>>> plt.show()
```

|  |  |
| --- | --- |
| pareto(a, size=None) | source code |

```
Draw samples from a Pareto distribution with specified shape.

This is a simplified version of the Generalized Pareto distribution
(available in SciPy), with the scale set to one and the location set to
zero. Most authors default the location to one.

The Pareto distribution must be greater than zero, and is unbounded above.
It is also known as the "80-20 rule".  In this distribution, 80 percent of
the weights are in the lowest 20 percent of the range, while the other 20
percent fill the remaining 80 percent of the range.

Parameters
----------
shape : float, > 0.
    Shape of the distribution.
size : tuple of ints
    Output shape.  If the given shape is, e.g., ``(m, n, k)``, then
    ``m * n * k`` samples are drawn.

See Also
--------
scipy.stats.distributions.genpareto.pdf : probability density function,
    distribution or cumulative density function, etc.

Notes
-----
The probability density for the Pareto distribution is

.. math:: p(x) = \frac{am^a}{x^{a+1}}

where :math:`a` is the shape and :math:`m` the location

The Pareto distribution, named after the Italian economist Vilfredo Pareto,
is a power law probability distribution useful in many real world problems.
Outside the field of economics it is generally referred to as the Bradford
distribution. Pareto developed the distribution to describe the
distribution of wealth in an economy.  It has also found use in insurance,
web page access statistics, oil field sizes, and many other problems,
including the download frequency for projects in Sourceforge [1].  It is
one of the so-called "fat-tailed" distributions.


References
----------
.. [1] Francis Hunt and Paul Johnson, On the Pareto Distribution of
       Sourceforge projects.
.. [2] Pareto, V. (1896). Course of Political Economy. Lausanne.
.. [3] Reiss, R.D., Thomas, M.(2001), Statistical Analysis of Extreme
       Values, Birkhauser Verlag, Basel, pp 23-30.
.. [4] Wikipedia, "Pareto distribution",
       http://en.wikipedia.org/wiki/Pareto_distribution

Examples
--------
Draw samples from the distribution:

>>> a, m = 3., 1. # shape and mode
>>> s = np.random.pareto(a, 1000) + m

Display the histogram of the samples, along with
the probability density function:

>>> import matplotlib.pyplot as plt
>>> count, bins, ignored = plt.hist(s, 100, normed=True, align='center')
>>> fit = a*m**a/bins**(a+1)
>>> plt.plot(bins, max(count)*fit/max(fit),linewidth=2, color='r')
>>> plt.show()
```

|  |  |
| --- | --- |
| permutation(x) | source code |

```
Randomly permute a sequence, or return a permuted range.

Parameters
----------
x : int or array_like
    If `x` is an integer, randomly permute ``np.arange(x)``.
    If `x` is an array, make a copy and shuffle the elements
    randomly.

Returns
-------
out : ndarray
    Permuted sequence or array range.

Examples
--------
>>> np.random.permutation(10)
array([1, 7, 4, 3, 0, 9, 2, 5, 8, 6])

>>> np.random.permutation([1, 4, 9, 12, 15])
array([15,  1,  9,  4, 12])
```

|  |  |
| --- | --- |
| poisson(lam=1.0, size=None) | source code |

```
Draw samples from a Poisson distribution.

The Poisson distribution is the limit of the Binomial
distribution for large N.

Parameters
----------
lam : float
    Expectation of interval, should be >= 0.
size : int or tuple of ints, optional
    Output shape. If the given shape is, e.g., ``(m, n, k)``, then
    ``m * n * k`` samples are drawn.

Notes
-----
The Poisson distribution

.. math:: f(k; \lambda)=\frac{\lambda^k e^{-\lambda}}{k!}

For events with an expected separation :math:`\lambda` the Poisson
distribution :math:`f(k; \lambda)` describes the probability of
:math:`k` events occurring within the observed interval :math:`\lambda`.

References
----------
.. [1] Weisstein, Eric W. "Poisson Distribution." From MathWorld--A Wolfram
       Web Resource. http://mathworld.wolfram.com/PoissonDistribution.html
.. [2] Wikipedia, "Poisson distribution",
   http://en.wikipedia.org/wiki/Poisson_distribution

Examples
--------
Draw samples from the distribution:

>>> import numpy as np
>>> s = np.random.poisson(5, 10000)

Display histogram of the sample:

>>> import matplotlib.pyplot as plt
>>> count, bins, ignored = plt.hist(s, 14, normed=True)
>>> plt.show()
```

|  |  |
| --- | --- |
| power(a, size=None) | source code |

```
Draws samples in [0, 1] from a power distribution with positive
exponent a - 1.

Also known as the power function distribution.

Parameters
----------
a : float
    parameter, > 0
size : tuple of ints
    Output shape.  If the given shape is, e.g., ``(m, n, k)``, then
            ``m * n * k`` samples are drawn.

Returns
-------
samples : {ndarray, scalar}
    The returned samples lie in [0, 1].

Raises
------
ValueError
    If a<1.

Notes
-----
The probability density function is

.. math:: P(x; a) = ax^{a-1}, 0 \le x \le 1, a>0.

The power function distribution is just the inverse of the Pareto
distribution. It may also be seen as a special case of the Beta
distribution.

It is used, for example, in modeling the over-reporting of insurance
claims.

References
----------
.. [1] Christian Kleiber, Samuel Kotz, "Statistical size distributions
       in economics and actuarial sciences", Wiley, 2003.
.. [2] Heckert, N. A. and Filliben, James J. (2003). NIST Handbook 148:
       Dataplot Reference Manual, Volume 2: Let Subcommands and Library
       Functions", National Institute of Standards and Technology Handbook
       Series, June 2003.
       http://www.itl.nist.gov/div898/software/dataplot/refman2/auxillar/powpdf.pdf

Examples
--------
Draw samples from the distribution:

>>> a = 5. # shape
>>> samples = 1000
>>> s = np.random.power(a, samples)

Display the histogram of the samples, along with
the probability density function:

>>> import matplotlib.pyplot as plt
>>> count, bins, ignored = plt.hist(s, bins=30)
>>> x = np.linspace(0, 1, 100)
>>> y = a*x**(a-1.)
>>> normed_y = samples*np.diff(bins)[0]*y
>>> plt.plot(x, normed_y)
>>> plt.show()

Compare the power function distribution to the inverse of the Pareto.

>>> from scipy import stats
>>> rvs = np.random.power(5, 1000000)
>>> rvsp = np.random.pareto(5, 1000000)
>>> xx = np.linspace(0,1,100)
>>> powpdf = stats.powerlaw.pdf(xx,5)

>>> plt.figure()
>>> plt.hist(rvs, bins=50, normed=True)
>>> plt.plot(xx,powpdf,'r-')
>>> plt.title('np.random.power(5)')

>>> plt.figure()
>>> plt.hist(1./(1.+rvsp), bins=50, normed=True)
>>> plt.plot(xx,powpdf,'r-')
>>> plt.title('inverse of 1 + np.random.pareto(5)')

>>> plt.figure()
>>> plt.hist(1./(1.+rvsp), bins=50, normed=True)
>>> plt.plot(xx,powpdf,'r-')
>>> plt.title('inverse of stats.pareto(5)')
```

|  |  |
| --- | --- |
| rand(d0, d1, dn, ...) | source code |

```
Random values in a given shape.

Create an array of the given shape and propagate it with
random samples from a uniform distribution
over ``[0, 1)``.

Parameters
----------
d0, d1, ..., dn : int
    Shape of the output.

Returns
-------
out : ndarray, shape ``(d0, d1, ..., dn)``
    Random values.

See Also
--------
random

Notes
-----
This is a convenience function. If you want an interface that
takes a shape-tuple as the first argument, refer to
`random`.

Examples
--------
>>> np.random.rand(3,2)
array([[ 0.14022471,  0.96360618],  #random
       [ 0.37601032,  0.25528411],  #random
       [ 0.49313049,  0.94909878]]) #random
```

|  |  |
| --- | --- |
| randint(low, high=None, size=None) | source code |

```
Return random integers from `low` (inclusive) to `high` (exclusive).

Return random integers from the "discrete uniform" distribution in the
"half-open" interval [`low`, `high`). If `high` is None (the default),
then results are from [0, `low`).

Parameters
----------
low : int
    Lowest (signed) integer to be drawn from the distribution (unless
    ``high=None``, in which case this parameter is the *highest* such
    integer).
high : int, optional
    If provided, one above the largest (signed) integer to be drawn
    from the distribution (see above for behavior if ``high=None``).
size : int or tuple of ints, optional
    Output shape. Default is None, in which case a single int is
    returned.

Returns
-------
out : int or ndarray of ints
    `size`-shaped array of random integers from the appropriate
    distribution, or a single such random int if `size` not provided.

See Also
--------
random.random_integers : similar to `randint`, only for the closed
    interval [`low`, `high`], and 1 is the lowest value if `high` is
    omitted. In particular, this other one is the one to use to generate
    uniformly distributed discrete non-integers.

Examples
--------
>>> np.random.randint(2, size=10)
array([1, 0, 0, 0, 1, 1, 0, 0, 1, 0])
>>> np.random.randint(1, size=10)
array([0, 0, 0, 0, 0, 0, 0, 0, 0, 0])

Generate a 2 x 4 array of ints between 0 and 4, inclusive:

>>> np.random.randint(5, size=(2, 4))
array([[4, 0, 2, 1],
       [3, 2, 2, 0]])
```

|  |  |
| --- | --- |
| randn(d1=..., dn=..., ...) | source code |

```
Return a sample (or samples) from the "standard normal" distribution.

If positive, int_like or int-convertible arguments are provided,
`randn` generates an array of shape ``(d1, ..., dn)``, filled
with random floats sampled from a univariate "normal" (Gaussian)
distribution of mean 0 and variance 1 (if any of the :math:`d_i` are
floats, they are first converted to integers by truncation). A single
float randomly sampled from the distribution is returned if no
argument is provided.

This is a convenience function.  If you want an interface that takes a
tuple as the first argument, use `numpy.random.standard_normal` instead.

Parameters
----------
d1, ..., dn : `n` ints, optional
    The dimensions of the returned array, should be all positive.

Returns
-------
Z : ndarray or float
    A ``(d1, ..., dn)``-shaped array of floating-point samples from
    the standard normal distribution, or a single such float if
    no parameters were supplied.

See Also
--------
random.standard_normal : Similar, but takes a tuple as its argument.

Notes
-----
For random samples from :math:`N(\mu, \sigma^2)`, use:

``sigma * np.random.randn(...) + mu``

Examples
--------
>>> np.random.randn()
2.1923875335537315 #random

Two-by-four array of samples from N(3, 6.25):

>>> 2.5 * np.random.randn(2, 4) + 3
array([[-4.49401501,  4.00950034, -1.81814867,  7.29718677],  #random
       [ 0.39924804,  4.68456316,  4.99394529,  4.84057254]]) #random
```

|  |  |
| --- | --- |
| random(size=None) | source code |

```
Return random floats in the half-open interval [0.0, 1.0).

Results are from the "continuous uniform" distribution over the
stated interval.  To sample :math:`Unif[a, b), b > a` multiply
the output of `random_sample` by `(b-a)` and add `a`::

  (b - a) * random_sample() + a

Parameters
----------
size : int or tuple of ints, optional
    Defines the shape of the returned array of random floats. If None
    (the default), returns a single float.

Returns
-------
out : float or ndarray of floats
    Array of random floats of shape `size` (unless ``size=None``, in which
    case a single float is returned).

Examples
--------
>>> np.random.random_sample()
0.47108547995356098
>>> type(np.random.random_sample())
<type 'float'>
>>> np.random.random_sample((5,))
array([ 0.30220482,  0.86820401,  0.1654503 ,  0.11659149,  0.54323428])

Three-by-two array of random numbers from [-5, 0):

>>> 5 * np.random.random_sample((3, 2)) - 5
array([[-3.99149989, -0.52338984],
       [-2.99091858, -0.79479508],
       [-1.23204345, -1.75224494]])
```

|  |  |
| --- | --- |
| random\_integers(low, high=None, size=None) | source code |

```
Return random integers between `low` and `high`, inclusive.

Return random integers from the "discrete uniform" distribution in the
closed interval [`low`, `high`].  If `high` is None (the default),
then results are from [1, `low`].

Parameters
----------
low : int
    Lowest (signed) integer to be drawn from the distribution (unless
    ``high=None``, in which case this parameter is the *highest* such
    integer).
high : int, optional
    If provided, the largest (signed) integer to be drawn from the
    distribution (see above for behavior if ``high=None``).
size : int or tuple of ints, optional
    Output shape. Default is None, in which case a single int is returned.

Returns
-------
out : int or ndarray of ints
    `size`-shaped array of random integers from the appropriate
    distribution, or a single such random int if `size` not provided.

See Also
--------
random.randint : Similar to `random_integers`, only for the half-open
    interval [`low`, `high`), and 0 is the lowest value if `high` is
    omitted.

Notes
-----
To sample from N evenly spaced floating-point numbers between a and b,
use::

  a + (b - a) * (np.random.random_integers(N) - 1) / (N - 1.)

Examples
--------
>>> np.random.random_integers(5)
4
>>> type(np.random.random_integers(5))
<type 'int'>
>>> np.random.random_integers(5, size=(3.,2.))
array([[5, 4],
       [3, 3],
       [4, 5]])

Choose five random numbers from the set of five evenly-spaced
numbers between 0 and 2.5, inclusive (*i.e.*, from the set
:math:`{0, 5/8, 10/8, 15/8, 20/8}`):

>>> 2.5 * (np.random.random_integers(5, size=(5,)) - 1) / 4.
array([ 0.625,  1.25 ,  0.625,  0.625,  2.5  ])

Roll two six sided dice 1000 times and sum the results:

>>> d1 = np.random.random_integers(1, 6, 1000)
>>> d2 = np.random.random_integers(1, 6, 1000)
>>> dsums = d1 + d2

Display results as a histogram:

>>> import matplotlib.pyplot as plt
>>> count, bins, ignored = plt.hist(dsums, 11, normed=True)
>>> plt.show()
```

|  |  |
| --- | --- |
| random\_sample(size=None) | source code |

```
Return random floats in the half-open interval [0.0, 1.0).

Results are from the "continuous uniform" distribution over the
stated interval.  To sample :math:`Unif[a, b), b > a` multiply
the output of `random_sample` by `(b-a)` and add `a`::

  (b - a) * random_sample() + a

Parameters
----------
size : int or tuple of ints, optional
    Defines the shape of the returned array of random floats. If None
    (the default), returns a single float.

Returns
-------
out : float or ndarray of floats
    Array of random floats of shape `size` (unless ``size=None``, in which
    case a single float is returned).

Examples
--------
>>> np.random.random_sample()
0.47108547995356098
>>> type(np.random.random_sample())
<type 'float'>
>>> np.random.random_sample((5,))
array([ 0.30220482,  0.86820401,  0.1654503 ,  0.11659149,  0.54323428])

Three-by-two array of random numbers from [-5, 0):

>>> 5 * np.random.random_sample((3, 2)) - 5
array([[-3.99149989, -0.52338984],
       [-2.99091858, -0.79479508],
       [-1.23204345, -1.75224494]])
```

|  |  |
| --- | --- |
| ranf(size=None) | source code |

```
Return random floats in the half-open interval [0.0, 1.0).

Results are from the "continuous uniform" distribution over the
stated interval.  To sample :math:`Unif[a, b), b > a` multiply
the output of `random_sample` by `(b-a)` and add `a`::

  (b - a) * random_sample() + a

Parameters
----------
size : int or tuple of ints, optional
    Defines the shape of the returned array of random floats. If None
    (the default), returns a single float.

Returns
-------
out : float or ndarray of floats
    Array of random floats of shape `size` (unless ``size=None``, in which
    case a single float is returned).

Examples
--------
>>> np.random.random_sample()
0.47108547995356098
>>> type(np.random.random_sample())
<type 'float'>
>>> np.random.random_sample((5,))
array([ 0.30220482,  0.86820401,  0.1654503 ,  0.11659149,  0.54323428])

Three-by-two array of random numbers from [-5, 0):

>>> 5 * np.random.random_sample((3, 2)) - 5
array([[-3.99149989, -0.52338984],
       [-2.99091858, -0.79479508],
       [-1.23204345, -1.75224494]])
```

|  |  |
| --- | --- |
| rayleigh(scale=1.0, size=None) | source code |

```
Draw samples from a Rayleigh distribution.

The :math:`\chi` and Weibull distributions are generalizations of the
Rayleigh.

Parameters
----------
scale : scalar
    Scale, also equals the mode. Should be >= 0.
size : int or tuple of ints, optional
    Shape of the output. Default is None, in which case a single
    value is returned.

Notes
-----
The probability density function for the Rayleigh distribution is

.. math:: P(x;scale) = \frac{x}{scale^2}e^{\frac{-x^2}{2 \cdotp scale^2}}

The Rayleigh distribution arises if the wind speed and wind direction are
both gaussian variables, then the vector wind velocity forms a Rayleigh
distribution. The Rayleigh distribution is used to model the expected
output from wind turbines.

References
----------
..[1] Brighton Webs Ltd., Rayleigh Distribution,
      http://www.brighton-webs.co.uk/distributions/rayleigh.asp
..[2] Wikipedia, "Rayleigh distribution"
      http://en.wikipedia.org/wiki/Rayleigh_distribution

Examples
--------
Draw values from the distribution and plot the histogram

>>> values = hist(np.random.rayleigh(3, 100000), bins=200, normed=True)

Wave heights tend to follow a Rayleigh distribution. If the mean wave
height is 1 meter, what fraction of waves are likely to be larger than 3
meters?

>>> meanvalue = 1
>>> modevalue = np.sqrt(2 / np.pi) * meanvalue
>>> s = np.random.rayleigh(modevalue, 1000000)

The percentage of waves larger than 3 meters is:

>>> 100.*sum(s>3)/1000000.
0.087300000000000003
```

|  |  |
| --- | --- |
| sample(size=None) | source code |

```
Return random floats in the half-open interval [0.0, 1.0).

Results are from the "continuous uniform" distribution over the
stated interval.  To sample :math:`Unif[a, b), b > a` multiply
the output of `random_sample` by `(b-a)` and add `a`::

  (b - a) * random_sample() + a

Parameters
----------
size : int or tuple of ints, optional
    Defines the shape of the returned array of random floats. If None
    (the default), returns a single float.

Returns
-------
out : float or ndarray of floats
    Array of random floats of shape `size` (unless ``size=None``, in which
    case a single float is returned).

Examples
--------
>>> np.random.random_sample()
0.47108547995356098
>>> type(np.random.random_sample())
<type 'float'>
>>> np.random.random_sample((5,))
array([ 0.30220482,  0.86820401,  0.1654503 ,  0.11659149,  0.54323428])

Three-by-two array of random numbers from [-5, 0):

>>> 5 * np.random.random_sample((3, 2)) - 5
array([[-3.99149989, -0.52338984],
       [-2.99091858, -0.79479508],
       [-1.23204345, -1.75224494]])
```

|  |  |
| --- | --- |
| seed(seed=None) | source code |

```
Seed the generator.

This method is called when `RandomState` is initialized. It can be
called again to re-seed the generator. For details, see `RandomState`.

Parameters
----------
seed : int or array_like, optional
    Seed for `RandomState`.

See Also
--------
RandomState
```

|  |  |
| --- | --- |
| set\_state(state) | source code |

```
Set the internal state of the generator from a tuple.

For use if one has reason to manually (re-)set the internal state of the
"Mersenne Twister"[1]_ pseudo-random number generating algorithm.

Parameters
----------
state : tuple(str, ndarray of 624 uints, int, int, float)
    The `state` tuple has the following items:

    1. the string 'MT19937', specifying the Mersenne Twister algorithm.
    2. a 1-D array of 624 unsigned integers ``keys``.
    3. an integer ``pos``.
    4. an integer ``has_gauss``.
    5. a float ``cached_gaussian``.

Returns
-------
out : None
    Returns 'None' on success.

See Also
--------
get_state

Notes
-----
`set_state` and `get_state` are not needed to work with any of the
random distributions in NumPy. If the internal state is manually altered,
the user should know exactly what he/she is doing.

For backwards compatibility, the form (str, array of 624 uints, int) is
also accepted although it is missing some information about the cached
Gaussian value: ``state = ('MT19937', keys, pos)``.

References
----------
.. [1] M. Matsumoto and T. Nishimura, "Mersenne Twister: A
   623-dimensionally equidistributed uniform pseudorandom number
   generator," *ACM Trans. on Modeling and Computer Simulation*,
   Vol. 8, No. 1, pp. 3-30, Jan. 1998.
```

|  |  |
| --- | --- |
| standard\_cauchy(size=None) | source code |

```
Standard Cauchy distribution with mode = 0.

Also known as the Lorentz distribution.

Parameters
----------
size : int or tuple of ints
    Shape of the output.

Returns
-------
samples : ndarray or scalar
    The drawn samples.

Notes
-----
The probability density function for the full Cauchy distribution is

.. math:: P(x; x_0, \gamma) = \frac{1}{\pi \gamma \bigl[ 1+
          (\frac{x-x_0}{\gamma})^2 \bigr] }

and the Standard Cauchy distribution just sets :math:`x_0=0` and
:math:`\gamma=1`

The Cauchy distribution arises in the solution to the driven harmonic
oscillator problem, and also describes spectral line broadening. It
also describes the distribution of values at which a line tilted at
a random angle will cut the x axis.

When studying hypothesis tests that assume normality, seeing how the
tests perform on data from a Cauchy distribution is a good indicator of
their sensitivity to a heavy-tailed distribution, since the Cauchy looks
very much like a Gaussian distribution, but with heavier tails.

References
----------
..[1] NIST/SEMATECH e-Handbook of Statistical Methods, "Cauchy
      Distribution",
      http://www.itl.nist.gov/div898/handbook/eda/section3/eda3663.htm
..[2] Weisstein, Eric W. "Cauchy Distribution." From MathWorld--A
      Wolfram Web Resource.
      http://mathworld.wolfram.com/CauchyDistribution.html
..[3] Wikipedia, "Cauchy distribution"
      http://en.wikipedia.org/wiki/Cauchy_distribution

Examples
--------
Draw samples and plot the distribution:

>>> s = np.random.standard_cauchy(1000000)
>>> s = s[(s>-25) & (s<25)]  # truncate distribution so it plots well
>>> plt.hist(s, bins=100)
>>> plt.show()
```

|  |  |
| --- | --- |
| standard\_exponential(size=None) | source code |

```
Draw samples from the standard exponential distribution.

`standard_exponential` is identical to the exponential distribution
with a scale parameter of 1.

Parameters
----------
size : int or tuple of ints
    Shape of the output.

Returns
-------
out : float or ndarray
    Drawn samples.

Examples
--------
Output a 3x8000 array:

>>> n = np.random.standard_exponential((3, 8000))
```

|  |  |
| --- | --- |
| standard\_gamma(shape, size=None) | source code |

```
Draw samples from a Standard Gamma distribution.

Samples are drawn from a Gamma distribution with specified parameters,
shape (sometimes designated "k") and scale=1.

Parameters
----------
shape : float
    Parameter, should be > 0.
size : int or tuple of ints
    Output shape.  If the given shape is, e.g., ``(m, n, k)``, then
    ``m * n * k`` samples are drawn.

Returns
-------
samples : ndarray or scalar
    The drawn samples.

See Also
--------
scipy.stats.distributions.gamma : probability density function,
    distribution or cumulative density function, etc.

Notes
-----
The probability density for the Gamma distribution is

.. math:: p(x) = x^{k-1}\frac{e^{-x/\theta}}{\theta^k\Gamma(k)},

where :math:`k` is the shape and :math:`\theta` the scale,
and :math:`\Gamma` is the Gamma function.

The Gamma distribution is often used to model the times to failure of
electronic components, and arises naturally in processes for which the
waiting times between Poisson distributed events are relevant.

References
----------
.. [1] Weisstein, Eric W. "Gamma Distribution." From MathWorld--A
       Wolfram Web Resource.
       http://mathworld.wolfram.com/GammaDistribution.html
.. [2] Wikipedia, "Gamma-distribution",
       http://en.wikipedia.org/wiki/Gamma-distribution

Examples
--------
Draw samples from the distribution:

>>> shape, scale = 2., 1. # mean and width
>>> s = np.random.standard_gamma(shape, 1000000)

Display the histogram of the samples, along with
the probability density function:

>>> import matplotlib.pyplot as plt
>>> import scipy.special as sps
>>> count, bins, ignored = plt.hist(s, 50, normed=True)
>>> y = bins**(shape-1) * ((np.exp(-bins/scale))/ \
...                       (sps.gamma(shape) * scale**shape))
>>> plt.plot(bins, y, linewidth=2, color='r')
>>> plt.show()
```

|  |  |
| --- | --- |
| standard\_normal(size=None) | source code |

```
Returns samples from a Standard Normal distribution (mean=0, stdev=1).

Parameters
----------
size : int or tuple of ints, optional
    Output shape. Default is None, in which case a single value is
    returned.

Returns
-------
out : float or ndarray
    Drawn samples.

Examples
--------
>>> s = np.random.standard_normal(8000)
>>> s
array([ 0.6888893 ,  0.78096262, -0.89086505, ...,  0.49876311, #random
       -0.38672696, -0.4685006 ])                               #random
>>> s.shape
(8000,)
>>> s = np.random.standard_normal(size=(3, 4, 2))
>>> s.shape
(3, 4, 2)
```

|  |  |
| --- | --- |
| standard\_t(df, size=None) | source code |

```
Standard Student's t distribution with df degrees of freedom.

A special case of the hyperbolic distribution.
As `df` gets large, the result resembles that of the standard normal
distribution (`standard_normal`).

Parameters
----------
df : int
    Degrees of freedom, should be > 0.
size : int or tuple of ints, optional
    Output shape. Default is None, in which case a single value is
    returned.

Returns
-------
samples : ndarray or scalar
    Drawn samples.

Notes
-----
The probability density function for the t distribution is

.. math:: P(x, df) = \frac{\Gamma(\frac{df+1}{2})}{\sqrt{\pi df}
          \Gamma(\frac{df}{2})}\Bigl( 1+\frac{x^2}{df} \Bigr)^{-(df+1)/2}

The t test is based on an assumption that the data come from a Normal
distribution. The t test provides a way to test whether the sample mean
(that is the mean calculated from the data) is a good estimate of the true
mean.

The derivation of the t-distribution was forst published in 1908 by William
Gisset while working for the Guinness Brewery in Dublin. Due to proprietary
issues, he had to publish under a pseudonym, and so he used the name
Student.

References
----------
.. [1] Dalgaard, Peter, "Introductory Statistics With R",
       Springer, 2002.
.. [2] Wikipedia, "Student's t-distribution"
       http://en.wikipedia.org/wiki/Student's_t-distribution

Examples
--------
From Dalgaard page 83 [1]_, suppose the daily energy intake for 11
women in Kj is:

>>> intake = np.array([5260., 5470, 5640, 6180, 6390, 6515, 6805, 7515, \
...                    7515, 8230, 8770])

Does their energy intake deviate systematically from the recommended
value of 7725 kJ?

We have 10 degrees of freedom, so is the sample mean within 95% of the
recommended value?

>>> s = np.random.standard_t(10, size=100000)
>>> np.mean(intake)
6753.636363636364
>>> intake.std(ddof=1)
1142.1232221373727

Calculate the t statistic, setting the ddof parameter to the unbiased
value so the divisor in the standard deviation will be degrees of
freedom, N-1.

>>> t = (np.mean(intake)-7725)/(intake.std(ddof=1)/np.sqrt(len(intake)))
>>> import matplotlib.pyplot as plt
>>> h = plt.hist(s, bins=100, normed=True)

For a one-sided t-test, how far out in the distribution does the t
statistic appear?

>>> >>> np.sum(s<t) / float(len(s))
0.0090699999999999999  #random

So the p-value is about 0.009, which says the null hypothesis has a
probability of about 99% of being true.
```

|  |  |
| --- | --- |
| triangular(left, mode, right, size=None) | source code |

```
Draw samples from the triangular distribution.

The triangular distribution is a continuous probability distribution with
lower limit left, peak at mode, and upper limit right. Unlike the other
distributions, these parameters directly define the shape of the pdf.

Parameters
----------
left : scalar
    Lower limit.
mode : scalar
    The value where the peak of the distribution occurs.
    The value should fulfill the condition ``left <= mode <= right``.
right : scalar
    Upper limit, should be larger than `left`.
size : int or tuple of ints, optional
    Output shape. Default is None, in which case a single value is
    returned.

Returns
-------
samples : ndarray or scalar
    The returned samples all lie in the interval [left, right].

Notes
-----
The probability density function for the Triangular distribution is

.. math:: P(x;l, m, r) = \begin{cases}
          \frac{2(x-l)}{(r-l)(m-l)}& \text{for $l \leq x \leq m$},\\
          \frac{2(m-x)}{(r-l)(r-m)}& \text{for $m \leq x \leq r$},\\
          0& \text{otherwise}.
          \end{cases}

The triangular distribution is often used in ill-defined problems where the
underlying distribution is not known, but some knowledge of the limits and
mode exists. Often it is used in simulations.

References
----------
..[1] Wikipedia, "Triangular distribution"
      http://en.wikipedia.org/wiki/Triangular_distribution

Examples
--------
Draw values from the distribution and plot the histogram:

>>> import matplotlib.pyplot as plt
>>> h = plt.hist(np.random.triangular(-3, 0, 8, 100000), bins=200,
...              normed=True)
>>> plt.show()
```

|  |  |
| --- | --- |
| uniform(low=0.0, high=1.0, size=1) | source code |

```
Draw samples from a uniform distribution.

Samples are uniformly distributed over the half-open interval
``[low, high)`` (includes low, but excludes high).  In other words,
any value within the given interval is equally likely to be drawn
by `uniform`.

Parameters
----------
low : float, optional
    Lower boundary of the output interval.  All values generated will be
    greater than or equal to low.  The default value is 0.
high : float
    Upper boundary of the output interval.  All values generated will be
    less than high.  The default value is 1.0.
size : tuple of ints, int, optional
    Shape of output.  If the given size is, for example, (m,n,k),
    m*n*k samples are generated.  If no shape is specified, a single sample
    is returned.

Returns
-------
out : ndarray
    Drawn samples, with shape `size`.

See Also
--------
randint : Discrete uniform distribution, yielding integers.
random_integers : Discrete uniform distribution over the closed interval
                  ``[low, high]``.
random_sample : Floats uniformly distributed over ``[0, 1)``.
random : Alias for `random_sample`.
rand : Convenience function that accepts dimensions as input, e.g.,
       ``rand(2,2)`` would generate a 2-by-2 array of floats, uniformly
       distributed over ``[0, 1)``.

Notes
-----
The probability density function of the uniform distribution is

.. math:: p(x) = \frac{1}{b - a}

anywhere within the interval ``[a, b)``, and zero elsewhere.

Examples
--------
Draw samples from the distribution:

>>> s = np.random.uniform(-1,0,1000)

All values are within the given interval:

>>> np.all(s >= -1)
True

>>> np.all(s < 0)
True

Display the histogram of the samples, along with the
probability density function:

>>> import matplotlib.pyplot as plt
>>> count, bins, ignored = plt.hist(s, 15, normed=True)
>>> plt.plot(bins, np.ones_like(bins), linewidth=2, color='r')
>>> plt.show()
```

|  |  |
| --- | --- |
| vonmises(mu=0.0, kappa=1.0, size=None) | source code |

```
Draw samples from a von Mises distribution.

Samples are drawn from a von Mises distribution with specified mode (mu)
and dispersion (kappa), on the interval [-pi, pi].

The von Mises distribution (also known as the circular normal
distribution) is a continuous probability distribution on the circle. It
may be thought of as the circular analogue of the normal distribution.

Parameters
----------
mu : float
    Mode ("center") of the distribution.
kappa : float, >= 0.
    Dispersion of the distribution.
size : {tuple, int}
    Output shape.  If the given shape is, e.g., ``(m, n, k)``, then
    ``m * n * k`` samples are drawn.

Returns
-------
samples : {ndarray, scalar}
    The returned samples live on the unit circle [-\pi, \pi].

See Also
--------
scipy.stats.distributions.vonmises : probability density function,
    distribution or cumulative density function, etc.

Notes
-----
The probability density for the von Mises distribution is

.. math:: p(x) = \frac{e^{\kappa cos(x-\mu)}}{2\pi I_0(\kappa)},

where :math:`\mu` is the mode and :math:`\kappa` the dispersion,
and :math:`I_0(\kappa)` is the modified Bessel function of order 0.

The von Mises, named for Richard Edler von Mises, born in
Austria-Hungary, in what is now the Ukraine. He fled to the United
States in 1939 and became a professor at Harvard. He worked in
probability theory, aerodynamics, fluid mechanics, and philosophy of
science.

References
----------
.. [1] Abramowitz, M. and Stegun, I. A. (ed.), Handbook of Mathematical
       Functions, National Bureau of Standards, 1964; reprinted Dover
       Publications, 1965.
.. [2] von Mises, Richard, 1964, Mathematical Theory of Probability
       and Statistics (New York: Academic Press).
.. [3] Wikipedia, "Von Mises distribution",
       http://en.wikipedia.org/wiki/Von_Mises_distribution

Examples
--------
Draw samples from the distribution:

>>> mu, kappa = 0.0, 4.0 # mean and dispersion
>>> s = np.random.vonmises(mu, kappa, 1000)

Display the histogram of the samples, along with
the probability density function:

>>> import matplotlib.pyplot as plt
>>> import scipy.special as sps
>>> count, bins, ignored = plt.hist(s, 50, normed=True)
>>> x = np.arange(-np.pi, np.pi, 2*np.pi/50.)
>>> y = -np.exp(kappa*np.cos(x-mu))/(2*np.pi*sps.jn(0,kappa))
>>> plt.plot(x, y/max(y), linewidth=2, color='r')
>>> plt.show()
```

|  |  |
| --- | --- |
| wald(mean, scale, size=None) | source code |

```
Draw samples from a Wald, or Inverse Gaussian, distribution.

As the scale approaches infinity, the distribution becomes more like a
Gaussian.

Some references claim that the Wald is an Inverse Gaussian with mean=1, but
this is by no means universal.

The Inverse Gaussian distribution was first studied in relationship to
Brownian motion. In 1956 M.C.K. Tweedie used the name Inverse Gaussian
because there is an inverse relationship between the time to cover a unit
distance and distance covered in unit time.

Parameters
----------
mean : scalar
    Distribution mean, should be > 0.
scale : scalar
    Scale parameter, should be >= 0.
size : int or tuple of ints, optional
    Output shape. Default is None, in which case a single value is
    returned.

Returns
-------
samples : ndarray or scalar
    Drawn sample, all greater than zero.

Notes
-----
The probability density function for the Wald distribution is

.. math:: P(x;mean,scale) = \sqrt{\frac{scale}{2\pi x^3}}e^
                            \frac{-scale(x-mean)^2}{2\cdotp mean^2x}

As noted above the Inverse Gaussian distribution first arise from attempts
to model Brownian Motion. It is also a competitor to the Weibull for use in
reliability modeling and modeling stock returns and interest rate
processes.

References
----------
..[1] Brighton Webs Ltd., Wald Distribution,
      http://www.brighton-webs.co.uk/distributions/wald.asp
..[2] Chhikara, Raj S., and Folks, J. Leroy, "The Inverse Gaussian
      Distribution: Theory : Methodology, and Applications", CRC Press,
      1988.
..[3] Wikipedia, "Wald distribution"
      http://en.wikipedia.org/wiki/Wald_distribution

Examples
--------
Draw values from the distribution and plot the histogram:

>>> import matplotlib.pyplot as plt
>>> h = plt.hist(np.random.wald(3, 2, 100000), bins=200, normed=True)
>>> plt.show()
```

|  |  |
| --- | --- |
| weibull(a, size=None) | source code |

```
Weibull distribution.

Draw samples from a 1-parameter Weibull distribution with the given
shape parameter.

.. math:: X = (-ln(U))^{1/a}

Here, U is drawn from the uniform distribution over (0,1].

The more common 2-parameter Weibull, including a scale parameter
:math:`\lambda` is just :math:`X = \lambda(-ln(U))^{1/a}`.

The Weibull (or Type III asymptotic extreme value distribution for smallest
values, SEV Type III, or Rosin-Rammler distribution) is one of a class of
Generalized Extreme Value (GEV) distributions used in modeling extreme
value problems.  This class includes the Gumbel and Frechet distributions.

Parameters
----------
a : float
    Shape of the distribution.
size : tuple of ints
    Output shape.  If the given shape is, e.g., ``(m, n, k)``, then
    ``m * n * k`` samples are drawn.

See Also
--------
scipy.stats.distributions.weibull : probability density function,
    distribution or cumulative density function, etc.

gumbel, scipy.stats.distributions.genextreme

Notes
-----
The probability density for the Weibull distribution is

.. math:: p(x) = \frac{a}
                 {\lambda}(\frac{x}{\lambda})^{a-1}e^{-(x/\lambda)^a},

where :math:`a` is the shape and :math:`\lambda` the scale.

The function has its peak (the mode) at
:math:`\lambda(\frac{a-1}{a})^{1/a}`.

When ``a = 1``, the Weibull distribution reduces to the exponential
distribution.

References
----------
.. [1] Waloddi Weibull, Professor, Royal Technical University, Stockholm,
       1939 "A Statistical Theory Of The Strength Of Materials",
       Ingeniorsvetenskapsakademiens Handlingar Nr 151, 1939,
       Generalstabens Litografiska Anstalts Forlag, Stockholm.
.. [2] Waloddi Weibull, 1951 "A Statistical Distribution Function of Wide
       Applicability",  Journal Of Applied Mechanics ASME Paper.
.. [3] Wikipedia, "Weibull distribution",
       http://en.wikipedia.org/wiki/Weibull_distribution

Examples
--------
Draw samples from the distribution:

>>> a = 5. # shape
>>> s = np.random.weibull(a, 1000)

Display the histogram of the samples, along with
the probability density function:

>>> import matplotlib.pyplot as plt
>>> x = np.arange(1,100.)/50.
>>> def weib(x,n,a):
...     return (a / n) * (x / n)**(a - 1) * np.exp(-(x / n)**a)

>>> count, bins, ignored = plt.hist(np.random.weibull(5.,1000))
>>> x = np.arange(1,100.)/50.
>>> scale = count.max()/weib(x, 1., 5.).max()
>>> plt.plot(x, weib(x, 1., 5.)*scale)
>>> plt.show()
```

|  |  |
| --- | --- |
| zipf(a, size=None) | source code |

```
Draw samples from a Zipf distribution.

Samples are drawn from a Zipf distribution with specified parameter (a),
where a > 1.

The zipf distribution (also known as the zeta
distribution) is a continuous probability distribution that satisfies
Zipf's law, where the frequency of an item is inversely proportional to
its rank in a frequency table.

Parameters
----------
a : float
    parameter, > 1.
size : {tuple, int}
    Output shape.  If the given shape is, e.g., ``(m, n, k)``, then
    ``m * n * k`` samples are drawn.

Returns
-------
samples : {ndarray, scalar}
    The returned samples are greater than or equal to one.

See Also
--------
scipy.stats.distributions.zipf : probability density function,
    distribution or cumulative density function, etc.

Notes
-----
The probability density for the Zipf distribution is

.. math:: p(x) = \frac{x^{-a}}{\zeta(a)},

where :math:`\zeta` is the Riemann Zeta function.

Named after the American linguist George Kingsley Zipf, who noted that
the frequency of any word in a sample of a language is inversely
proportional to its rank in the frequency table.


References
----------
.. [1] Weisstein, Eric W. "Zipf Distribution." From MathWorld--A Wolfram
       Web Resource. http://mathworld.wolfram.com/ZipfDistribution.html
.. [2] Wikipedia, "Zeta distribution",
       http://en.wikipedia.org/wiki/Zeta_distribution
.. [3] Wikipedia, "Zipf's Law",
       http://en.wikipedia.org/wiki/Zipf%27s_law
.. [4] Zipf, George Kingsley (1932): Selected Studies of the Principle
       of Relative Frequency in Language. Cambridge (Mass.).

Examples
--------
Draw samples from the distribution:

>>> a = 2. # parameter
>>> s = np.random.zipf(a, 1000)

Display the histogram of the samples, along with
the probability density function:

>>> import matplotlib.pyplot as plt
>>> import scipy.special as sps
Truncate s values at 50 so plot is interesting
>>> count, bins, ignored = plt.hist(s[s<50], 50, normed=True)
>>> x = np.arange(1., 50.)
>>> y = x**(-a)/sps.zetac(a)
>>> plt.plot(x, y/max(y), linewidth=2, color='r')
>>> plt.show()
```

  


|  |  |
| --- | --- |
| Variables Details | [hide private] |

### ScalarType

Value:
:   ```
    (<type 'int'>,
     <type 'float'>,
     <type 'complex'>,
     <type 'long'>,
     <type 'bool'>,
     <type 'str'>,
     <type 'unicode'>,
     <type 'buffer'>,
    ...
    ```

### cast

Value:
:   ```
    {<type 'numpy.int64'>: <function <lambda> at 0x10a8bb0>, <type 'numpy.
    int16'>: <function <lambda> at 0x10a8bf0>, <type 'numpy.complex128'>: 
    <function <lambda> at 0x10a8c30>, <type 'numpy.uint64'>: <function <la
    mbda> at 0x10a8c70>, <type 'numpy.complex256'>: <function <lambda> at 
    0x10a8cf0>, <type 'numpy.float32'>: <function <lambda> at 0x10a8d30>, 
    <type 'numpy.bool_'>: <function <lambda> at 0x10a8cb0>, <type 'numpy.u
    int8'>: <function <lambda> at 0x10a8db0>, <type 'numpy.int32'>: <funct
    ion <lambda> at 0x10a8eb0>, <type 'numpy.int8'>: <function <lambda> at
    ...
    ```

### colorbar\_doc

Value:
:   ```
    '''

    Add a colorbar to a plot.

    Function signatures for the :mod:`~matplotlib.pyplot` interface; all
    but the first are also method signatures for the
    :meth:`~matplotlib.figure.Figure.colorbar` method::

    ...
    ```

### curve\_list

Value:
:   ```
    {'EP-C': <class 'PyDSTool.PyCont.Continuation.EquilibriumCurve'>,
     'FP-C': <class 'PyDSTool.PyCont.Continuation.FixedPointCurve'>,
     'H-C1': <class 'PyDSTool.PyCont.Continuation.HopfCurveOne'>,
     'H-C2': <class 'PyDSTool.PyCont.Continuation.HopfCurveTwo'>,
     'LC-C': <class 'PyDSTool.PyCont.Continuation.LimitCycleCurve'>,
     'LP-C': <class 'PyDSTool.PyCont.Continuation.FoldCurve'>,
     'UD-C': <class 'PyDSTool.PyCont.Continuation.UserDefinedCurve'>}
    ```

### index\_exp

Value:
:   ```
    <numpy.lib.index_tricks.IndexExpression object at 0x11543b0>
    ```

### nbytes

Value:
:   ```
    {<type 'numpy.int64'>: 8, <type 'numpy.int16'>: 2, <type 'numpy.comple
    x128'>: 16, <type 'numpy.uint64'>: 8, <type 'numpy.bool_'>: 1, <type '
    numpy.complex256'>: 32, <type 'numpy.float32'>: 4, <type 'numpy.int8'>
    : 1, <type 'numpy.uint8'>: 1, <type 'numpy.uint16'>: 2, <type 'numpy.o
    bject_'>: 4, <type 'numpy.float64'>: 8, <type 'numpy.int32'>: 4, <type
     'numpy.string_'>: 0, <type 'numpy.void'>: 0, <type 'numpy.float128'>:
     16, <type 'numpy.int32'>: 4, <type 'numpy.uint32'>: 4, <type 'numpy.u
    nicode_'>: 0, <type 'numpy.complex64'>: 8, <type 'numpy.uint32'>: 4}
    ```

### rcParams

Value:
:   ```
    {'figure.subplot.right': 0.90000000000000002, 'mathtext.cal': 'cursive
    ', 'font.fantasy': ['Comic Sans MS', 'Chicago', 'Charcoal', 'ImpactWes
    tern', 'fantasy'], 'xtick.minor.pad': 4, 'tk.pythoninspect': False, 'i
    mage.aspect': 'equal', 'font.cursive': ['Apple Chancery', 'Textile', '
    Zapf Chancery', 'Sand', 'cursive'], 'figure.subplot.hspace': 0.2000000
    0000000001, 'xtick.direction': 'in', 'axes.facecolor': 'w', 'mathtext.
    fontset': 'cm', 'ytick.direction': 'in', 'axes.axisbelow': False, 'lin
    es.markersize': 6, 'figure.dpi': 100.0, 'text.usetex': True, 'text.fon
    ...
    ```

### rcParamsDefault

Value:
:   ```
    {'figure.subplot.right': 0.90000000000000002, 'mathtext.cal': 'cursive
    ', 'font.fantasy': ['Comic Sans MS', 'Chicago', 'Charcoal', 'ImpactWes
    tern', 'fantasy'], 'xtick.minor.pad': 4, 'tk.pythoninspect': False, 'i
    mage.aspect': 'equal', 'font.cursive': ['Apple Chancery', 'Textile', '
    Zapf Chancery', 'Sand', 'cursive'], 'figure.subplot.hspace': 0.2000000
    0000000001, 'xtick.direction': 'in', 'axes.facecolor': 'w', 'mathtext.
    fontset': 'cm', 'ytick.direction': 'in', 'axes.axisbelow': False, 'lin
    es.markersize': 6, 'figure.dpi': 80, 'text.usetex': False, 'text.fonta
    ...
    ```

### sctypeDict

Value:
:   ```
    {0: <type 'numpy.bool_'>,
     1: <type 'numpy.int8'>,
     2: <type 'numpy.uint8'>,
     3: <type 'numpy.int16'>,
     4: <type 'numpy.uint16'>,
     5: <type 'numpy.int32'>,
     6: <type 'numpy.uint32'>,
     7: <type 'numpy.int32'>,
    ...
    ```

### sctypeNA

Value:
:   ```
    {'?': 'Bool',
     'B': 'UInt8',
     'Bool': <type 'numpy.bool_'>,
     'Complex128': <type 'numpy.complex256'>,
     'Complex32': <type 'numpy.complex64'>,
     'Complex64': <type 'numpy.complex128'>,
     'D': 'Complex64',
     'F': 'Complex32',
    ...
    ```

### sctypes

Value:
:   ```
    {'complex': [<type 'numpy.complex64'>,
                 <type 'numpy.complex128'>,
                 <type 'numpy.complex256'>],
     'float': [<type 'numpy.float32'>,
               <type 'numpy.float64'>,
               <type 'numpy.float128'>],
     'int': [<type 'numpy.int8'>,
             <type 'numpy.int16'>,
    ...
    ```

### typeDict

Value:
:   ```
    {0: <type 'numpy.bool_'>,
     1: <type 'numpy.int8'>,
     2: <type 'numpy.uint8'>,
     3: <type 'numpy.int16'>,
     4: <type 'numpy.uint16'>,
     5: <type 'numpy.int32'>,
     6: <type 'numpy.uint32'>,
     7: <type 'numpy.int32'>,
    ...
    ```

### typeNA

Value:
:   ```
    {'?': 'Bool',
     'B': 'UInt8',
     'Bool': <type 'numpy.bool_'>,
     'Complex128': <type 'numpy.complex256'>,
     'Complex32': <type 'numpy.complex64'>,
     'Complex64': <type 'numpy.complex128'>,
     'D': 'Complex64',
     'F': 'Complex32',
    ...
    ```

### typecodes

Value:
:   ```
    {'All': '?bhilqpBHILQPfdgFDGSUVOMm',
     'AllFloat': 'fdgFDG',
     'AllInteger': 'bBhHiIlLqQpP',
     'Character': 'c',
     'Complex': 'FDG',
     'Datetime': 'Mm',
     'Float': 'fdg',
     'Integer': 'bhilqp',
    ...
    ```

  


| Home | Trees | Indices | Help | PyDSTool |
| --- | --- | --- | --- | --- |

|  |  |
| --- | --- |
| Generated by Epydoc 3.0.1 on Fri May 4 15:24:03 2012 | http://epydoc.sourceforge.net |
